# Sex‐Specific Gene Expression Ontogeny During Gonadal Development in Post‐Metamorphic *Xenopus tropicalis*

**DOI:** 10.1002/mrd.70098

**Published:** 2026-04-06

**Authors:** Daniele Marini, Mauricio Roza, Cecilia Berg, Vanessa Brouard

**Affiliations:** ^1^ Department of Organismal Biology Uppsala University Uppsala Sweden; ^2^ Department of Veterinary Medicine University of Perugia Perugia Italy; ^3^ Science for Life Laboratory, Department of Environmental Science Stockholm University Stockholm Sweden

**Keywords:** Amphibia, germ cells, histology, pre‐pubertal development, tropical clawed frog

## Abstract

Sexual differentiation in amphibians involves dynamic coordination between germ cells, somatic tissues, and gene expression. However, post‐metamorphic gonadal maturation and sex‐specific transcriptional programs underlying it remain poorly characterized. In this study, pre‐pubertal gonadal development was analyzed in *Xenopus tropicalis* by integrating morphological, histological, and gene expression analyses. Froglets were sampled weekly from one until 8 weeks post‐metamorphosis. Morphometric and histological assessments revealed progressive gonadal maturation in both sexes. In males, testicular growth correlated with body size, whereas in females, ovarian development occurred independently of somatic parameters. Expression profiling of 12 genes showed sex‐specific mRNA patterns: *cyp17*, *amh*, and *amhr2* displayed a male‐biased expression correlated with testicular development, while *aldh1a2* and *ddx4* were preferentially upregulated in females and associated with ovarian growth and follicular oocyte number. *Id4* declined in females but remained stable in males, reflecting, together with *ddx4* expression, distinct pregametogenesis and germline maintenance strategies. Opposite regulation of *aldh1a2* and *cyp26b1* supported a transient meiotic wave in females versus its basal maintenance in males. Multivariate and network analyses highlighted *dmrt1*, *amhr2*, *cyp17*, *esr1*, and *ddx4* as potential nodes of sex‐specific regulatory networks. These findings reveal post‐metamorphic, sex‐specific transcriptional programs, and provide integrated molecular and histological endpoints for studies on vertebrate sex differentiation.

## Graphical abstract

Post‐metamorphic gonadal development in *Xenopus tropicalis* was analyzed through integrated morphometric, histological, and transcriptional approaches. Males showed upregulation of *cyp17*, *amh*, and *amhr2*, whereas females exhibited an increased *aldh1a2* and *ddx4*. *id4* and *ddx4* marked distinct germline states, and opposite *aldh1a2*‐*cyp26b1* regulation indicated sex‐specific retinoic acid pathway dynamics.

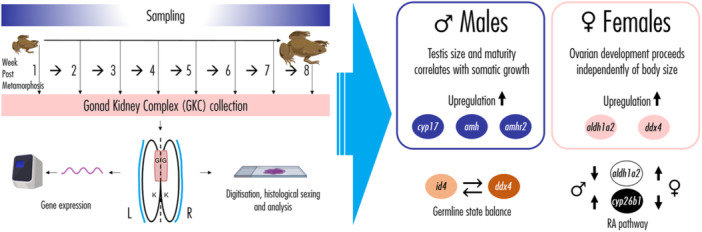

## 1 Introduction

Gonad differentiation is a developmental process common to all vertebrates. It involves sex determination in the undifferentiated gonad followed by sexual differentiation, when sex‐specific structures and molecular profiles arise (Nagahama et al. 2021). In most vertebrates, as well as in anuran amphibians, sex determination typically involves genetic and hormonal mechanisms (Capel 2017; Nagahama et al. 2021).

To study sexual determination and differentiation in vertebrates, and particularly in amphibians, *Xenopus laevis* (Daudin 1802) and *Xenopus (Silurana) tropicalis* (Gray 1864)—or African clawed frog and tropical clawed frog, respectively—have been used for decades in research. These species are easy to maintain and to breed all year round in captivity, which allows major findings in developmental biology (Hirsch et al. 2002; Tadjuidje and Heasman 2010). While both species share conserved features such as a comparable hypothalamus‐pituitary‐gonadal axis and Müllerian duct development—relevant to mammals and absent in fish models—, *X. tropicalis* offers specific advantages (Berg 2019; Berg et al. 2009): a shorter life cycle and a diploid genome. Moreover, the well‐defined larval developmental stages, the Nieuwkoop and Faber (NF) stages, of *X. laevis* can be adapted to the congeneric anuran model (Nieuwkoop and Faber 1956; Zahn et al. 2022).

In *X. laevis* tadpoles, sex‐determination takes place during NF50–NF52 (Yoshimoto et al. 2008), while the gonads are still undifferentiated between NF49 and NF53 (Piprek et al. 2017). In this species, females are heterogametic (ZW/ZZ sex chromosome system), and ovarian fate is triggered by *dm‐w* (*doublesex and mab3 related transcription factor W* – Yoshimoto et al. 2008), which antagonizes *dmrt1* (*doublesex and mab‐3 related transcription factor 1*), a male‐promoting factor essential for sex determination and testis development (Yoshimoto et al. 2010). By contrast, *X. tropicalis* lacks *dm‐w* and carries three sex chromosomes (Z, W, and a young Y) with heterogametic conditions in both sexes (Bewick et al. 2011; Roco et al. 2015; Roco et al. 2021; Yoshimoto et al. 2008): YZ, YW, or ZZ males and ZW or WW females. In this species, a male‐determining factor on the Y chromosome (presumably involving *dmrt1* or a related element) is dominant over the Z, whereas a W chromosome can feminize Z‐bearing individuals (Furman et al. 2020; Roco et al. 2015, 2021).

Histology for phenotypical sexing is widely used in clawed frogs before puberty occurs. In *X. laevis*, gonads diverge morphologically from NF53 with ovarian cortex–medulla separation and intramedullary lumen formation (secondary ovarian cavity), while male gonads develop testis cords (Piprek et al. 2017). At NF62, oocytes entering meiosis I are found in the ovaries, whereas the testes do not contain meiotic cells at this stage (Piprek et al. 2018). Similarly, in *X. tropicalis*, the histological distinction between male and female gonads becomes apparent after NF52 (El Jamil et al. 2008b), and is fully evident at NF58 (Haselman et al. 2015). Male gonads show early signs of differentiation beginning at NF48 with primordial germ cells (PGCs) migration that leads to the eventual disappearance of the cortex (El Jamil et al. 2008b; Haselman et al. 2015; Ogielska 2009), whereas female differentiation follows at NF51 with cortical PGCs retention and ovarian lumen formation by NF59 (El Jamil et al. 2008b; Ogielska 2009). By NF66, the final stage before metamorphosis, testicular cords in males are primordial and separated by the extracellular matrix; in females, ovaries display diplotene oocytes surrounded by follicular cells in the center and germ cell nests located at the periphery (El Jamil et al. 2008b).

Germline differentiation follows sexual differentiation, and in anurans, ovarian development mirrors mammals, with oocytes recruited from a limited oogonia pool formed during pre‐oogenesis in the tadpoles/juveniles (Ogielska et al. 2013; Ogielska et al. 2024). At metamorphosis, ovaries contain primary oogonia and early meiotic oocytes (Kvarnryd et al. 2011; Ogielska et al. 2024), which then progress through diplotene (when they arrest at meiosis I) to vitellogenic stages (Ogielska et al. 2024; Rasar and Hammes 2006). In *X. tropicalis* females, it has been shown that oocyte maturation starts at 16 weeks post‐metamorphosis (PM), associated with an increase in estradiol and vitellogenin concentrations in the blood, followed by an increase in pre‐vitellogenic and vitellogenic oocytes, in correlation with a significant gain in ovary and oviduct mass (Olmstead et al. 2009). Yet, based on the cellular composition of the gonad, females are only sexually mature around 26 weeks PM or ~7 months old (Olmstead et al. 2009). A shorter time has been observed in laboratory conditions, where females were able to reproduce at 6 months old (Hirsch et al. 2002). Moreover, studies from our laboratory have shown that female ovarian cell composition did not significantly differ between 4 and 8 weeks PM; however, the more mature germ cells observed were the Stages II pre‐vitellogenic oocytes (Svanholm et al. 2023; Svanholm et al. 2021). In male anurans, sexual differentiation includes prespermatogenesis in tadpoles, characteristic of gonocytes appearance and the establishment of the stem cell pool, followed by active spermatogenesis that initiates at the metamorphosis stage (Haczkiewicz et al. 2017). Spermatogenesis is marked by the progression of pale and dark spermatogonial stem cells (SSCs) toward secondary spermatogonia and then spermatocytes before morphological differentiation into spermatid and spermatozoa stages (Ogielska and Bartmanska 2009; Ogielska et al. 2024). In *X. tropicalis*, males are sexually mature around 20 weeks PM or ~5.5 months old (Olmstead et al. 2009); however, under laboratory conditions, males are sexually mature at 4 months old (Hirsch et al. 2002). Moreover, a precise description of the testis histological composition at 4 and 8 weeks PM revealed that spermatozoa are produced as early as 5 and 8 weeks after metamorphosis (Säfholm et al. 2016; Svanholm et al. 2023, 2021). These results indicate that major developmental events occur between metamorphosis and 8 weeks PM, but the precise timeline of juvenile germ cell differentiation and related gene expression in *X. tropicalis* remains unclear.

In *X. laevis*, sex‐specific gene expression appears as early as NF50, when the gonads are still undifferentiated (Piprek et al. 2018): females (ZW) show more dimorphic transcripts than males (ZZ), but from NF53 to NF62, males exhibit stronger dimorphism. In *X. tropicalis*, transcriptional divergence is limited at NF58 but becomes pronounced by NF66, when ovary‐enriched genes increase (coincident with oocytes entering meiosis I), whereas testis‐enriched genes are expressed earlier and remain relatively constant (Haselman et al. 2015).

The present study investigates the mRNA expression of genes involved in gonadal development during the pre‐pubertal period of *X. tropicalis*. It specifically addresses gaps concerning the timing of germ cell differentiation and sex‐specific gene expression in the early post‐metamorphic window, and the limited gene expression data available for this species.

The genes selected for the study are based on their developmental and functional roles in reproductive development and function in male and female vertebrates. The list is inspired by previous studies performed in *Xenopus* species (see Piprek et al. 2013, 2017, 2018) as well as in mammalian species. In particular, candidate genes, selected from amphibian and mammalian models, included sex‐differentiation genes (*dmrt1*, *cyp17* [*cytochrome P450 family 17 subfamily A member 1*], *cyp19* [*cytochrome P450 family 19 subfamily A member 1*], *amh* [*anti‐Müllerian hormone*] and *sox9* [*SRY‐box transcription factor 9*]*)*, germ cell genes (*id4* [*inhibitor of DNA binding 4*] *ddx4* [*DEAD‐box helicase 4*, also known as *vasa‐like gene 1* or *xvlg1*]), steroidogenic/signaling genes (*3βhsd* [*3 beta‐hydroxysteroid dehydrogenase*], *esr1* [*estrogen receptor 1*]), male‐specific genes (*amhr2* [*anti‐Müllerian hormone receptor 2*]), and retinoic acid pathway genes (*aldh1a2* [*aldehyde dehydrogenase 1 family member A2*], *cyp26b1* [*cytochrome P450 family 26 subfamily B member 1*]).

We hypothesize that the divergence in histological features and gene expression profiles between male and female gonads emerges during the pre‐pubertal period, coinciding with the onset of spermatogenesis in males and the early progression of oogenesis in females. This developmental window likely represents a critical transition in which sex‐specific molecular programs become aligned with the first histological signs of functional differentiation.

The aims were to (i) assess body growth, gonadal tissue development, and histological features, (ii) quantify the expression of selected genes, and (iii) integrate these data to clarify the chronology of sex‐specific gene expression and its association with gonadal development during the first 2 months PM, a key window for the onset of puberty in *X. tropicalis*.

## 2 Material and Methods

### 2.1 Animal Handling

*Xenopus tropicalis* tadpoles were generated from two different in‐lab mating pairs of adults. Adult frogs from Xenopus1 (Dexter, MI, USA) were prepared for mating by injection of human chorionic gonadotropin (0.9%) into the dorsal lymph sac (Pettersson et al. 2006). Seventy‐two hours after fertilization, tadpoles were arranged in five 15 L aquaria, each containing 60 tadpoles. During the weeks before metamorphosis, tadpoles were moved to new aquaria depending on the developmental stage to obtain a more homogeneous population in each tank. During the tadpole period, each aquarium was cleaned, and fresh water was added three times per week. Then, when the tadpoles completed metamorphosis (NF66), they were placed in flow‐through aquaria (20 L) and kept until the desired age. Water temperature, pH, and conductivity were monitored every day. Moreover, the nitrite and ammonia/ammonium levels were measured once a week, using standard kits from Sera (Gibbon, Sweden). The animals were kept in copper‐free tap water under a 12‐h light and 12‐h dark cycle. During the early stages, tadpoles were fed three times per day with Sera Micron (Sera, Heinsberg, Germany). From metamorphosis, the frogs were introduced progressively to Sera Vipan Baby food (Sera, Heinsberg, Germany) and Tropical Energy food (Aquatic Nature, Roeselare, Belgium), and Sera Micron was removed from their diet.

### 2.2 Dissection and Samplings

Between 5 and 10 animals were sampled weekly from 1 to 8 weeks PM. Based on age, four groups of metamorphosed frogs were established: Met 1‐2 (1–2 weeks PM), Met 3‐4 (3–4 weeks PM), Met 5‐6 (5–6 weeks PM), and Met 7‐8 (7–8 weeks PM). On the day of necropsy, the animal was anesthetized in 0.3% buffered MS‐222 solution (tricaine dissolved in sodium bicarbonate buffer, pH 7—Sigma Aldrich, Saint‐Louis, USA) before being euthanized by decapitation. For each animal, morphological parameters including body weight, body length, forelimb length, hindlimb length, liver weight, and fat body weight were measured. Liver and fat body somatic indices were calculated by dividing organ weight by body weight and multiplying by 100. The presence of nuptial pads was also recorded. Using a stereomicroscope, the right gonad‐kidney complex (GKC) was excised and snap‐frozen in liquid nitrogen for gene expression analysis, whereas the left GKC with the underlying skeletal and muscular tissue was fixed in 10% neutral buffered formalin (NBF) for histological evaluation.

### 2.3 Histological Processing and Analysis

The left GKC was immersed in 10% NBF for at least 48 h, and then transferred and stored in 70% ethanol until further processing. Tissues were dehydrated in increasing concentrations of ethanol (150 min in 96% ethanol and 50 min in 100% ethanol), followed by clarification in xylene (45 min) before infiltration in paraffin (low‐melting‐point paraffin, 52°C–54°C, for 50 min) ahead of the final step. Finally, the GKCs were embedded to obtain transverse sections. The tissues were initially sectioned at 10 µm and stained with toluidine blue to locate the gonads. Once the gonads were clearly visible, sections were reduced to 5 µm and used for haematoxylin‐eosin (HE) staining. For each animal, at least two sections 20 µm apart were stained with HE for phenotypical sex determination and evaluation of gonadal metrics and maturation.

The area of the gonad and the germ cell maturity were evaluated with NDP.view2 software (v2.8.24; Hamamatsu, Shizuoka, Japan). Testis maturity was determined using a scoring system based on detailed histological criteria of the male germ cell established in our laboratory (Svanholm et al. 2023). The maturity score was assigned to each testis according to the most advanced germ cell type present: 1—pale SSC, 2—dark SSC, 3—secondary spermatogonia, 4—primary spermatocyte, 5—secondary spermatocyte, 6—spermatid, 7—spermatozoa. Ovary maturation was assessed by counting the number of follicular oocytes (oocytes surrounded by a follicle cell layer) in a single ovary section. The histological scoring was performed by two operators using masked slide names.

### 2.4 Gene Expression Analysis

The right GKC of each individual was processed for gene expression analysis. Tissue homogenization was performed in two steps. First, the GKCs were fragmented using a sterile plastic pestle in a 1.5 mL tube initially dry, then with the addition of 100 µL of PureZOL (Biorad, Hercules, USA). In the second step, the homogenate was transferred to a 2 mL SafeSeal tube, where an additional 100 µL of PureZOL and titanium beads were added. Samples were then homogenized twice for 1 min at speed 8 using a Bullet Blender Storm 24 (NextAdvance, Troy, USA). After homogenization, RNA was extracted using the Aurum Total RNA Mini kit (BioRad, Hercules, USA), following the manufacturer's instructions. Briefly, 800 µL of PureZOL was added to each sample and incubated for 5 min at room temperature (RT). Then, 200 µL of chloroform was added to the lysate and the tube was vigorously shaken for 30 s. After 5 min of incubation at RT with regular mixing by inversion, the samples were centrifuged at 12,000 g for 15 min at 4°C. After centrifugation, the top aqueous phase was transferred to a new tube, and an equal volume of 70% ethanol was added and mixed thoroughly by pipetting. The lysate was then transferred onto the RNA‐binding column. Following centrifugation at 12,000 g for 1 min, the RNA column was washed with buffer solution before DNase I treatment. The DNase I solution mix was freshly prepared, as described by the manufacturer (Biorad, Hercules, USA), applied directly to the RNA column membrane, and incubated for 15 min. After DNase I treatment, the RNA column was washed twice before elution of the RNA via centrifugation with 30 µL of elution solution. Finally, RNA concentration and purity were assessed spectrophotometrically using a Spark microplate reader (Tecan, Männedorf, Switzerland), evaluating both the 260/280 nm and 230/280 nm absorbance ratios. Reverse transcription of RNA samples was performed with the iScript cDNA synthesis kit (BioRad, Hercules, USA). Five hundred nanograms of RNA were used for the reverse transcription into complementary DNA (cDNA). For each sample, 4 µL of iScript reaction mix and 1 µL of iScript reverse transcriptase were mixed with the RNA sample. To obtain the final volume of 20 µL, nuclease‐free water was added if necessary. BioRad Thermocycler T100 (Biorad, Hercules, USA) was used for the reverse transcription run with a protocol of 5 min at 25°C, 20 min at 46°C, and 1 min at 95°C. The cDNA sample concentration was adjusted to obtain 20 ng cDNA/µL for the quantitative polymerase chain reaction (qPCR). iQ SYBR Green Supermix (Biorad, Hercules, USA) was used in a final volume of 10 µL containing: 5 µL of SYBR Green Supermix, 0.3 µL of a 10 μM suspension of forward and reverse primers, 0.7 µL of water, and 4 µL of cDNA sample. The Biorad CFX384 instrument (Biorad, Hercules, USA) was used for the qPCR run with the following cycling conditions: 3 min at 95°C followed by 45 cycles of 10 s at 95°C and 30 s at 60°C (62°C were used for *cyp19* primers), and ending with the melting curve from 55°C up to 95°C with an increment of 0.5°C every 5 s. Each sample was analyzed in triplicate. The relative gene expression levels were calculated using the 2^−ΔCt^ method using the average of two housekeeping genes: *eukaryotic translation elongation factor 1 alpha 1* (*eef1α1*) and *ribosomal protein L8* (*rpl8*) (Orton et al. 2018; Verbrugghe et al. 2019) (Table 1). For each set of primers, amplification efficiency was determined using a standard curve generated from a cDNA mix of adult male and female GKC tissues. Six cDNA concentrations (ranging from 0.93 to 30 ng/µL) and a negative control with only water were used. The percentage of efficiency was calculated using the formula efficiency = (10^(−1/a)^−1) × 100, where “a” (slope) was derived from the linear regression of average Ct values plotted against the log of cDNA concentration. The primer sets used for this study showed efficiencies of 103.9% ± 12% for the male samples and 99.3% ± 13% for the female samples (see overall efficiency—average between sexes—in Table 1).

**Table 1:** qPCR primers sequence and average efficiency for *Xenopus tropicalis* candidate genes used in this study.

Gene name and symbol	Accession #	Forward sequence 5′ → 3′	Reverse sequence 5′ → 3′	Efficiency (%)
*eukaryotic translation elongation factor 1 alpha 1* (*eef1α1*)	NM_001016692.2	CTCTCAGGCTGACTGTGCTG	ATGCTCACGAGTTTGTCCGT	96
*ribosomal protein L8 (rpl8)* a	BC059744	CCCTCAACCATCAGGAGAGA	TCTTTGTACCACGCAGACGA	95
*SRY‐box transcription factor 9 (sox9)*	NM_001016853.2	ACATTCAGGTCAGTCCCAAGG	TCCCTCTTCAGGTCTGGCTT	99
*cytochrome P450 family 17 subfamily A member 1 (cyp17)*	NM_001127045.1	ATAAAGAGGCGTTTTGCGGC	AGTCCGACCTCCTGGGAAAT	97
*3 beta‐hydroxysteroid dehydrogenase (3βhsd)*	XM_031896427.1	CCAATCCCTGGTTACTAAGGCA	GTTTAGTGACTCCCCAATGGCA	107
*doublesex and mab‐3 related transcription factor 1 (dmrt1)*	XM_031890717.1	GGACAGAGTGTACCCAACCC	GAGAAAGCACTGCACTTGCC	116
*inhibitor of DNA binding 4 (id4)*	NM_001004839.1	CGCCGAACAAGAAAGTCAGC	CGGGTCAGTATTGAGGTCCG	98
*DEAD‐box helicase 4 (ddx4)*	NM_001016823.3	TGAAAGGGGTGGGCCTACTA	ATTCCAATCACCTCTTCCTCGAA	95
*cytochrome P450 family 19 subfamily A member 1 (cyp19)* b	NM_001097161.1	GAATCCCGTGCAGTATAACAGC	ACAGGTCTCCTCTTGATTCCATAG	129
*aldehyde dehydrogenase 1 family member A2* (*aldh1a2*)	NM_001045731.1	ATTGGCGTGTGTGGTCAGAT	ACAGGGCAGGAGCAATCTTC	113
*cytochrome P450 family 26 subfamily B member 1 (cyp26b1)*	NM_001079187.2	TCCCCTGTACAAAGCATAGACAA	GGTGTTACAATGCACTGCCC	88
*estrogen receptor 1 (esr1)*	NM_203535.1	AATGTGCCTCCAAGTCCTGT	TCTGTTGTCTGAACTTGACCTGT	112
*anti‐Müllerian hormone (amh)*	XM_004911423.4	GTCAGTCCAAGCTTTAGTACCCA	ATCGCCAACACTGTGATCGT	90
*anti‐Müllerian hormone receptor 2 (amhr2)* b	ENSXETT00000063675	AAAGACGGCCACAGCTTCC	CAGGATCCCAGCAATCTTCC	99

^a^ Orton et al. 2018.

^b^ Jansson et al. 2016.

### 2.5 Statistical Analysis

Morphological data, as well as testis maturity score, testis area, number of follicular oocytes, and ovary area, were compared between age groups using the Kruskal–Wallis test followed by Dunn's multiple comparisons test. Correlations between gonadal parameters and age, as well as body measurements and gene expression were assessed using the Pearson correlation coefficient using GraphPad Prism software (v5.0).

Alterations in gene expression associated with sex and age were analyzed in R (v4.4.0) using Generalized Linear Models (GLM) from the gamma family of distributions with a log link function. Pairwise comparisons of estimated marginal means were performed using the *emmeans* package (v1.10.2), with *p* values adjusted for multiple testing using the Holm method. Overall effects were evaluated through analysis of deviance (ANODEV), which assesses the significance of main factors (sex, developmental stage) and their interaction (sex × stage) within the GLM framework. Principal component analysis (PCA) was performed in R using the *FactoMineR* package (v2.11) and visualized with *factoextra* (v1.0.7), where missing values were imputed with the imputePCA function from the *missMDA* package (v1.19).

Bayesian network analysis was conducted in JASP (v.0.18.1) to evaluate conditional dependencies of transcripts with the Gaussian copula graphical model estimator. Networks were stratified by sex and by developmental stage, grouping individuals as younger (Met 1‐4) or older (Met 5‐8). A Markov Chain Monte Carlo approach was run for 10,000 iterations with a burn‐in of 5000 and a fixed seed of 1. The prior probability of edge inclusion was set to 0.5 with a G‐Wishart prior (df = 3). Edges were retained when the Bayes Factor (BF₁₀) was greater than 10. Outputs included network, edge evidence, and centrality plots, together with weight matrices and edge evidence probability tables.

Pairwise correlations between gene expression levels, pooling all stages and sexes, were assessed in JASP (v.0.18.1) using Spearman's rank correlation coefficient (*ρ*) with two‐sided significance testing (*p* < 0.05), and results were visualized as a heatmap.

## 3 Results

### 3.1 Morphological Data

**Figure 1:**
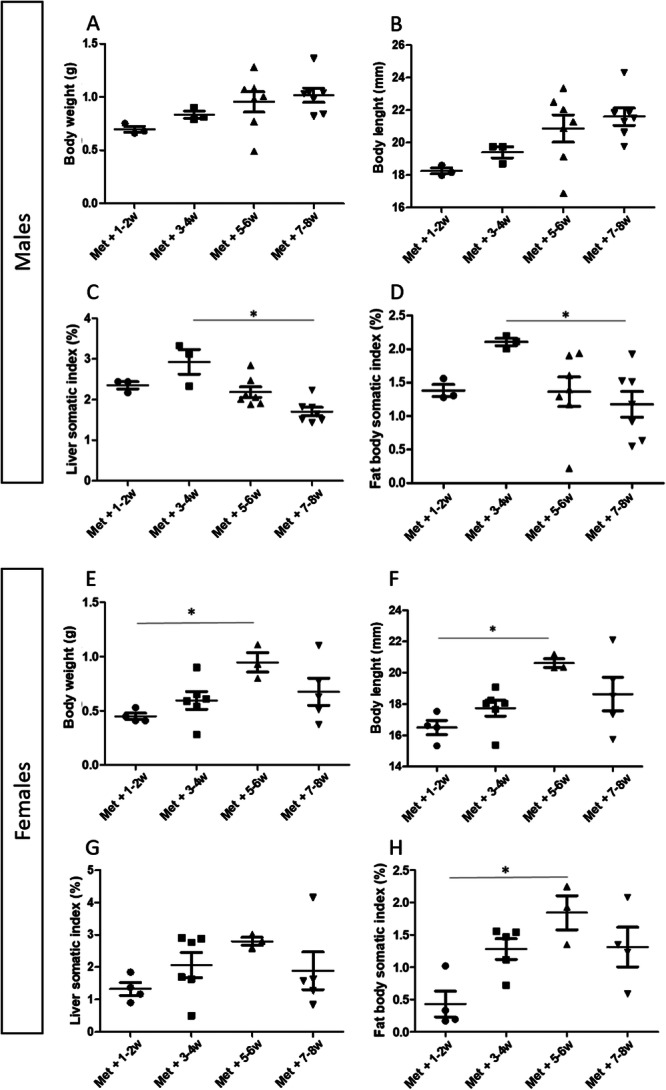
Body, liver, and fat body measurement in post‐metamorphic male and female frogs. (A) Male and (E) female body weight at different ages after metamorphosis. (B) Male and (F) female body length at different ages after metamorphosis. (C) Male and (G) female liver somatic index at different ages after metamorphosis. (D) Male and (H) female fat body somatic index at different ages after metamorphosis. N per sex and per post‐metamorphic age is: three males and four females at Met 1‐2, three males and six females at Met 3‐4, seven males and three females at Met 5‐6, and seven males and five females at Met 7‐8. Statistical significances were analyzed using the Kruskal–Wallis test followed by Dunn's comparison. Asterisks denote significance levels: *p < 0.05.

In males, both body weight and length tended to increase with age, although these changes were not statistically significant (Figure 1A,B). The liver and fat body somatic indices exhibited an initial increase from Met 1‐2 to Met 3‐4, followed by a significant decrease from Met 3‐4 to Met 7‐8 (*p* < 0.05, Figure 1C,D). Correlation analysis revealed that body weight, body length, forelimb length, and hindlimb length were strongly and significantly correlated with each other (*r* = 0.81–0.97, *p* < 0.0001, Table S1A), but none of these parameters correlated significantly with liver weight and fat body weight. Liver weight and fat body weight demonstrated a significant positive correlation (*r* = 0.75, *p* < 0.001) (Table S1A).

In females, morphological growth seems to occur slightly later and to peak at 5‐6 weeks PM of age (Met 5‐6). Specifically, body weight, body length, and fat body somatic index significantly increased from Met 1‐2 to Met 5‐6 (*p* < 0.05, Figure 1E,F,H), and tended to decrease at Met 7‐8. No significant differences were observed for the liver somatic index among age groups in females (Figure 1G). Moreover, most morphological parameters were significantly correlated with each other (*r* = 0.64–0.96, *p* < 0.01–0.0001, Table S1B), except for forelimb length, which did not correlate significantly with liver weight or fat body weight (Table S1B).

### 3.2 Histological Evaluation of the Gonads

**Figure 2:**
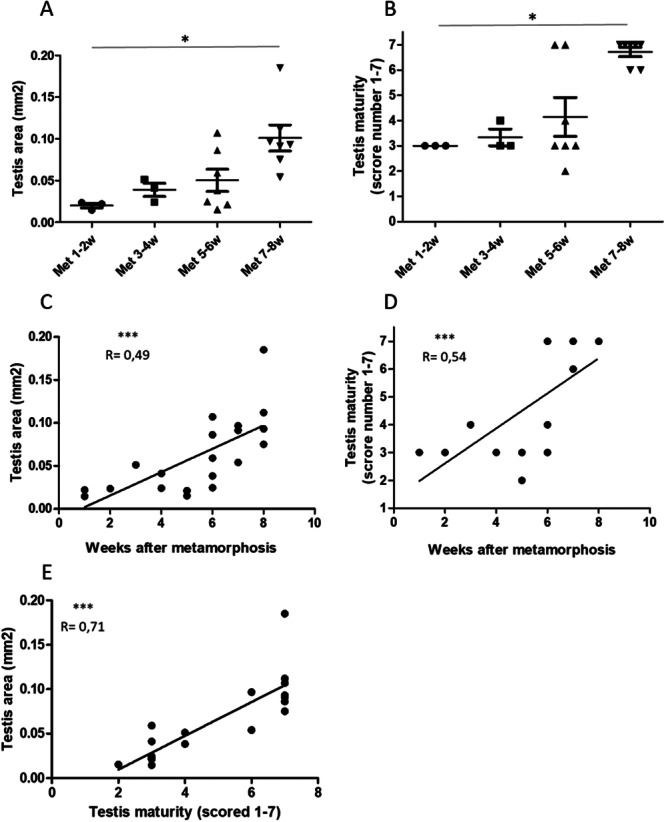
Histological analysis of male gonads in post‐metamorphic frogs. (A) Quantification of the testis area at different ages after metamorphosis. (B) Evaluation of testis maturity at different ages after metamorphosis. Testis maturity was defined with a scoring system assigned depending on the most mature germ cells observed: 1: Pale primary spermatogonia, 2: Dark primary spermatogonia, 3: Secondary spermatogonia, 4: Primary spermatocyte, 5: Secondary spermatocyte, 6: Spermatid, 7: Spermatozoa. (C) Correlation between the testis area and the frog age, (D) between the testis maturity and the ages, and between (E) the testis maturity and the testis area. N for males per post‐metamorphic age is: three males at Met 1‐2, three males at Met 3‐4, seven males at Met 5‐6, and seven males at Met 7‐8. Statistical significances were analyzed using the Kruskal–Wallis test followed by Dunn's comparison or Pearson correlation test with *p < 0.05 and ***p < 0.001.

The testis area and testis maturity score increased progressively with age in juvenile *X. tropicalis*, (*p* < 0.05, Figure 2A,B). Both testis area and testis maturity score showed a significant positive correlation with frog age (*r* = 0.49 and *r* = 0.54, respectively; *p* < 0.001, Figure 2C,D). Finally, testis area and testis maturity score displayed a significant positive correlation with each other (*r* = 0.71, *p* < 0.001; Figure 2E).

**Figure 3:**
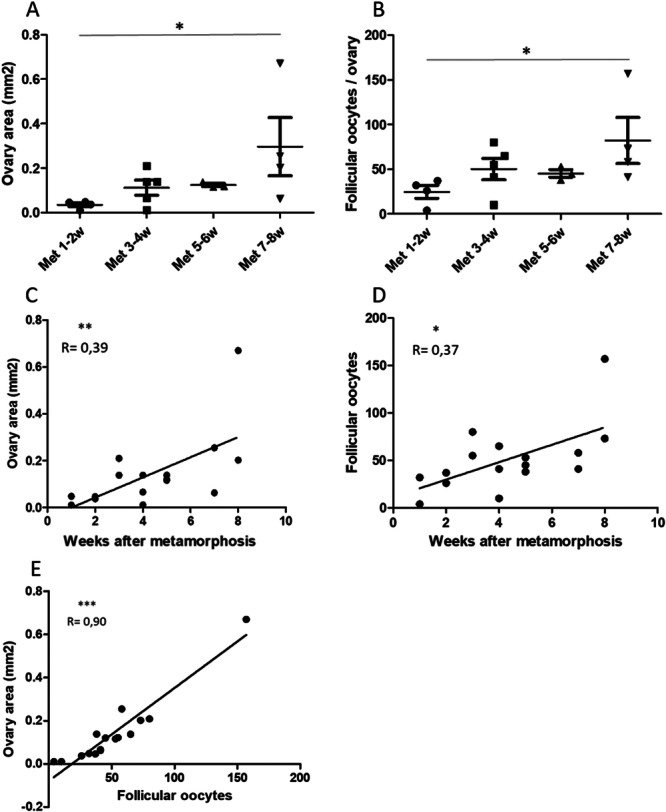
Histological analysis of female gonads in post‐metamorphic frogs. (A) Quantification of the ovary area at different ages after metamorphosis. (B) Number of follicular oocytes for the corresponding area at different ages after metamorphosis. (C) Correlation between the ovary area and the frog age, (D) between the number of follicular oocytes and the age, and between (E) the number of follicles and the ovary area. N for females per post‐metamorphic age is: four females at Met 1‐2, six females at Met 3‐4, three females at Met 5‐6, and five females at Met 7‐8. Statistical significances were analyzed using the Kruskall–Wallis test followed by Dunn's comparison or Pearson correlation test with *p < 0.05 and ***p < 0.001.

Ovary area and the number of follicular oocytes increased with age in juvenile *X. tropicalis* (*p* < 0.05; Figure 3A). In addition, both the ovarian area and the number of follicular oocytes demonstrated significant positive correlations with age (*r *= 0.39, *p* < 0.01, and *r* = 0.37, *p* < 0.05, respectively; Figure 3C,D). Furthermore, the ovary area and the number of follicular oocytes were significantly positively correlated with each other (*r* = 0.90; *p* < 0.001; Figure 3E).

Finally, gonadal development was positively correlated with growth in males but not in females. Specifically, testis area and testis maturity score were positively correlated with body weight, body length, hindlimb length, and forelimb length (*p* ≤ 0.05). Nonetheless, testis area and testis maturity score did not show significant correlations with liver weight and fat body weight (Table S2).

### 3.3 Gene Expression

Relative gene expression was analyzed from GKC samples of male and female frogs, from 1‐2 to 7‐8 weeks post‐metamorphosis.

The ANODEV supported sex‐ and stage‐dependent regulation of several transcripts (Table S3). A significant overall effect of sex was detected for *id4*, *ddx4*, *cyp17*, *amh*, *3βhsd*, *esr1*, *cyp26b1*, and *aldh1a2*, indicating that these genes were consistently expressed at different levels in males and females across all stages. Developmental stage alone significantly affected *ddx4*, *cyp17*, *amh*, *cyp19*, and *aldh1a2*, reflecting temporal modulation of their expression irrespective of sex. Significant sex × stage interactions emerged for *amhr2* and *aldh1a2*, showing that the magnitude and direction of sex differences varied depending on the developmental window. In contrast, no significant effects were observed for *dmrt1* and *sox9*.

**Figure 4:**
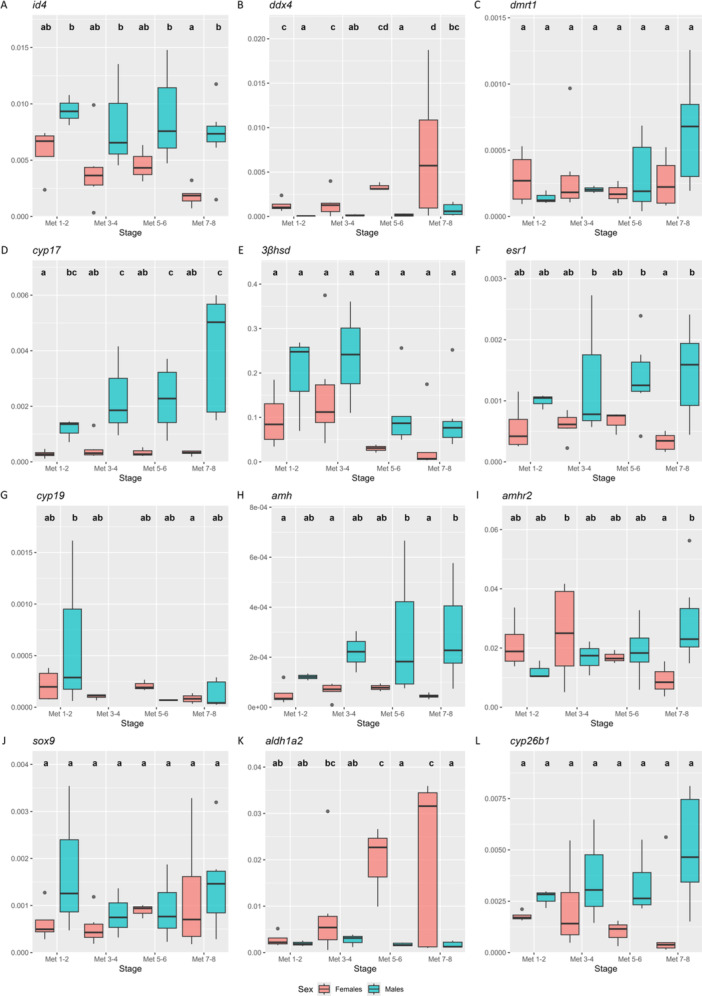
Gene expression ontogeny in the gonad‐kidney complex of post‐metamorphic frogs. Relative gene expression in the gonad kidney complex in males (blue boxes) and females (red boxes) from Met 1‐2 to Met 7‐8. Relative expression for germ cells specific genes: (A) *id4*, (B) *ddx4*, and (C) *dmrt1*; steroidogenic/signaling genes: (D) *cyp17*, (E) *3βhsd*, (F) *esr1*, and (G) *cyp19*; male differentiation genes: (H) *amh*, (I) *amhr2*, and (J) *sox9*; retinoic pathway genes: (K) *aldh1a2* and (L) *cyp26b1*. Statistical significance *p* < 0.05 is achieved when the above letters are different between the two groups.

The relative expression of the germ cell‐specific gene *id4* tended to—not significantly—decrease from Met 1‐2 to Met 7‐8 in females, whereas no significant variation was observed over time in males. At Met 7‐8 weeks, *id4* expression was significantly higher in males compared to females (Figure 4A). Relative *ddx4* expression significantly increased from Met 5‐6 to Met 7‐8 in males, and from Met 3‐4 to Met 7‐8 in females. In addition, *ddx4* expression remained significantly higher in females compared to males at all ages (Figure 4B). Analysis of *dmrt1* expression revealed no significant differences between sexes or across time points (Figure 4C). The expression of genes involved in steroidogenesis included *cyp17*, which tended to increase with time in male frogs from Met 1‐2 to Met 7‐8, whereas no change was observed over time in females. Moreover, *cyp17* expression remained significantly higher in males compared to females at all ages (Figure 4D). The relative expression of *3βhsd* (Figure 4E) did not differ between sexes or across age groups, while *cyp19* (Figure 4G) showed significantly higher expression in males at Met 1‐2 versus females at Met 7‐8. The gene *esr1* did not display significant variation across ages within sexes, but it was significantly higher in males at Met 7‐8 (Figure 4F).

For the genes implicated in male gonad differentiation, *amh* relative expression did not significantly change over time, but it was significantly higher in males compared to females at Met 7‐8 (Figure 4H). Expression of its receptor, *amhr2*, significantly decreased in females from Met 3‐4 to Met 7‐8, and no significant changes were observed in males. At Met 7‐8, *amhr2* levels were significantly lower in females compared to males (Figure 4I). Relative *sox9* expression in post‐metamorphic frogs did not show differences between sexes or age groups (Figure 4J).

Finally, for the genes implicated in the retinoic acid pathway, *aldh1a2* expression significantly increased in females from Met 1‐2 to Met 5‐6 and Met 7‐8, whereas no significant variation was observed in males. In addition, *aldh1a2* expression was significantly higher in females compared to males at Met 5‐6 and Met 7‐8 (Figure 4K). Lastly, *cyp26b1* gene expression did not significantly differ over time, nor did it differ between sexes (Figure 4L).

### 3.4 Principal Component Analysis

**Figure 5:**
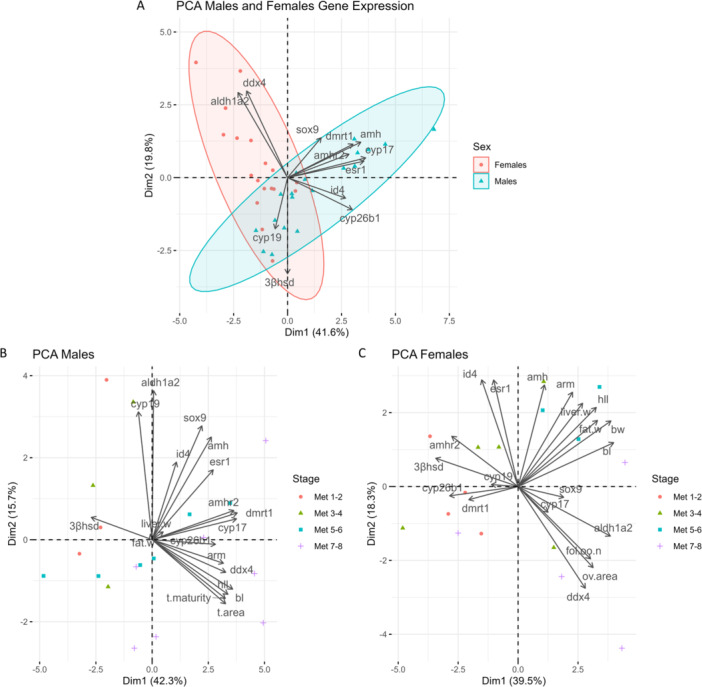
Principal component analysis (PCA) for sex‐specific gene expression in gonad/kidney complex, body measurements, and gonad maturity in post‐metamorphic frogs. (A) Principal component analysis representation for sex‐specific gene expression in the gonad/kidney complex in males (M, blue symbol) and females (F, red symbol) post‐metamorphic frogs. (B) PCA for post‐metamorphosis male frogs stratified by age with body measurements, gonad maturity and relative gene expression. (C) PCA for post‐metamorphic female frogs stratified by age with body measurements, gonad maturity and relative gene expression. Relative gene expression for germ cells genes: *id4*, *ddx4* and *dmrt1*; steroidogenic/signaling genes: *cyp17*, *3βhsd*, *cyp19* and *esr1*; male differentiation genes: *amh*, *amhr2*, *sox9*; retinoic pathway genes: *aldh1a2* and *cyp26b1*.

PCA based on sex‐specific gene expression in male and female frogs explained 61.4% of the variance with the first two dimensions (Figure 5A). It revealed a distinct clustering of female and male individuals, indicating a strong association between sex and relative gene expression. Genes associated with females were *ddx4*, and *aldh1a2*, whereas genes associated with male individuals were *cyp17*, *dmrt1*, *amh*, *amhr2, esr1*, *id4*, and *cyp26b1* (Figure 5A). PCA performed with males alone, with individuals grouped by age and incorporating gene expression and gonadal parameters, did not reveal strong associations, although it explained approximately 58% of the variance (Figure 5B). However, this PCA representation highlighted a strong association between body measurements and gonad maturity data with *ddx4*, and a negative association between *3βhsd* expression and *dmrt1*, *amh*, *amhr2*, *cyp17*, *cyp26b1*, and *ddx4* genes, indicated by arrows pointing in opposite directions (Figure 5B). PCA carried out on females alone—and explaining 57.8% of the variance—demonstrated a strong association between gonad maturity data and relative expression of *ddx4* and *aldh1a2* (Figure 5C).

### 3.5 Correlation Between Gene Expression and Gonadal Development

Correlations between relative gene expression of sex‐specific genes and gonad maturity data were analyzed in male and female post‐metamorphic frogs (Table 2). In males, testis area and testis maturity showed significant positive correlations with the relative expression of *ddx4*, *dmrt1*, *cyp17*, *amhr2*, and *cyp26b1*. In females, the ovarian area was positively correlated with the relative expression of *ddx4* and *aldh1a2* and negatively correlated with *amhr2* and *3βhsd*. The number of follicular oocytes was positively correlated with *ddx4* and *aldh1a2* (Table 2).

**Table 2:** Correlation between specific genes expression and gonad maturity in post‐metamorphic frogs.

	*id4*	*ddx4*	*dmrt1*	*sox9*	*cyp17*	*amh*	*amhr2*	*3βhsd*	*esr1*	*cyp19*	*cyp26b1*	*aldh1a2*
Testis area	−0.08	0.71***	0.61**	0.23	0.62**	0.45	0.49*	−0.39	0.16	−0.29	0.47*	−0.23
Testis maturity	−0.01	0.60**	0.66**	0.28	0.57*	0.44	0.58**	−0.43	0.15	−0.29	0.59**	−0.24
Ovary area	−0.47	0.92****	−0.36	0.19	0.12	−0.02	−0.52*	−0.54*	−0.34	−0.36	−0.46	0.75***
Follicular oocytes number	−0.42	0.86****	−0.36	0.21	0.14	0.08	−0.47	−0.49	−0.31	−0.32	−0.43	0.69**

*Note:* Correlation between relative gene expression in the gonad kidney complex and gonad histology in post‐metamorphic frogs. Relative expression for germ cells genes: *id4*, *ddx4* and *dmrt1*; steroidogenic/signaling genes: *cyp17*, *3βhsd*, *cyp19* and *esr1*; male differentiation genes: *amh*, *amhr2*, *sox9*; retinoic pathway genes: *aldh1a2* and *cyp26b1*. Numbers represent the *r* coefficient of Pearson correlation, and statistical significance level is denoted by asterisks: *p < 0.05, **p < 0.01, ***p < 0.001, ****p < 0.0001.

### 3.6 Bayesian Network Analysis

**Figure 6:**
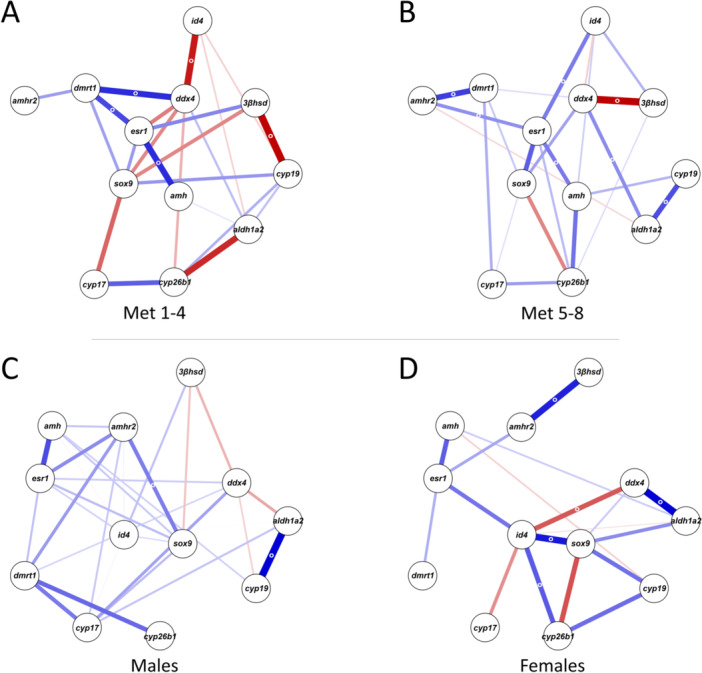
Bayesian network plots illustrating conditional dependencies between transcripts from the gonad/kidney complex in post‐metamorphic frogs, stratified by age (A, B) and sex (C, D). (A) Plot of relative gene expression data from younger froglets, spanning the 1st–4th weeks post‐metamorphosis (Met 1‐4). (B) Plot of mRNA data from older frogs, spanning the 5th–8th weeks post‐metamorphosis (Met 5‐8). (C) Plot based on relative gene expression data from male frogs. (D) Plot based on mRNA data from female frogs. The thickness and color of edges represent the strength/weight and direction of associations, respectively, with red edges indicating negative associations and blue edges indicating positive associations. Included edges (BF₁₀ > 10) are marked by “°” sign. Germ cells genes: *id4*, *ddx4* and *dmrt1*; steroidogenic/signaling genes: *cyp17*, *3βhsd*, *cyp19* and *esr1*; male differentiation genes: *amh*, *amhr2*, *sox9*; retinoic pathway genes: *aldh1a2* and *cyp26b1*.

The Bayesian network analysis identified a limited number of strong conditional dependencies among transcripts, with network topology varying by both developmental stage and sex. The network structure is illustrated in the network plots in Figure 6, edge evidence plots in Figure S1, and centrality plots in Figure S2, while weight matrices and edge evidence probability tables are presented in Table S4. Only connections with substantial evidence for inclusion (BF₁₀ > 10 – marked by the “°” sign in Figure 6 and colored in blue in Figure S1) are described here.

In early juveniles (Met 1‐4), seven nodes exhibited strong dependencies, and in three cases the transcripts showed two associations (Figure S1A). Specifically, *ddx4* was positively associated with *dmrt1* and negatively with *id4*, while *esr1* showed positive connections to both *dmrt1* and *amh*, and *3βhsd* was negatively associated with *cyp19* (Figure 6A). At later stages (Met 5‐8), nine nodes demonstrated well‐supported dependencies (Figure S1B), including three strong connections: two positive (*dmrt1‐amhr2* and *aldh1a2‐cyp19*) and one negative (*ddx4‐3βhsd*) (Figure 6B). The only connection scoring BF₁₀ > 10 retained across both Met 1‐4 and Met 5‐8 groups was *esr1‐amh*, which appeared to lose its positive dependence as age increased.

Stratification by sex revealed that in males, only four nodes exhibited strongly supported associations (Figure S1C), where the connection *aldh1a2‐cyp19* was markedly positive, while *amhr2‐sox9* showed a moderate positive association (Figure 6C). In the female group, seven nodes demonstrated robust associations (Figure S1D): three connections were clearly positive (*amhr2‐3βhsd*, *id4‐sox9*, and *ddx4‐aldh1a2*), one was moderately positive (*id4‐cyp26b1*), and one was negative (*id4‐ddx4*) (Figure 6D). No robust edges were retained across both male and female groups.

### 3.7 Correlations Between Gene Expression Levels

**Figure 7:**
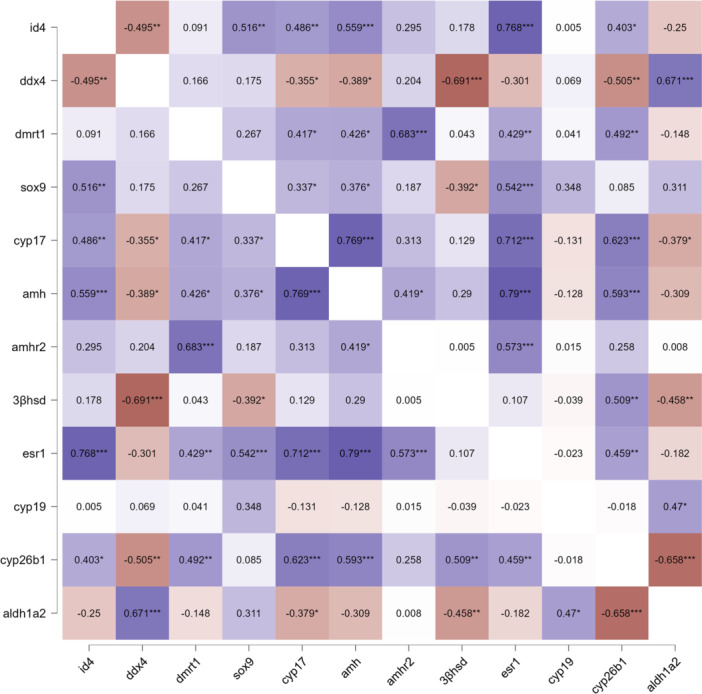
Heatmap of pairwise Spearman's rank correlations among transcript expression levels across developmental stages and sexes in post‐metamorphic *Xenopus tropicalis*. Blue squares indicate positive correlations and red squares negative correlations, with color intensity proportional to correlation strength. Numbers in each cell represent the correlation coefficient (*ρ*). Asterisks denote significance levels: *p < 0.05, **p < 0.01, ***p < 0.001. Germ cells genes: *id4*, *ddx4* and *dmrt1*; steroidogenic/signaling genes: *cyp17*, *3βhsd*, *cyp19* and *esr1*; male differentiation genes: *amh*, *amhr2*, *sox9*; retinoic pathway genes: *aldh1a2* and *cyp26b1*.

Pairwise Spearman's rank correlations among mRNA levels encompassing all stages and both sexes are shown in Figure 7. Several strong associations emerged, with multiple correlations reaching high statistical significance (***p < 0.001). Specifically, *amh* correlated positively with *cyp17* (*ρ* = 0.769), *cyp26b1* (*ρ* = 0.593), *id4* (*ρ* = 0.559), and *esr1* (*ρ* = 0.790). *amhr2* showed highly significant positive associations with *dmrt1* (*ρ* = 0.683) and *esr1* (*ρ* = 0.573). Likewise, *cyp17* correlated strongly with *cyp26b1* (*ρ* = 0.623) and *esr1* (*ρ* = 0.712). In addition, *aldh1a2* was positively correlated with *ddx4* (*ρ* = 0.671), but was negatively associated with *cyp26b1* (*ρ* = −0.658). *ddx4* further displayed negative correlations with *3βhsd* (*ρ* = −0.691). Among other highly significant relationships (*p* < 0.001), *esr1* correlated positively with both *id4* (*ρ* = 0.768) and *sox9* (*ρ* = 0.542).

## 4 Discussion

In this study, we investigated the post‐metamorphic development of *Xenopus tropicalis* by analyzing morphological parameters, gonadal maturation at the histological level, gene expression profiles, and network associations among key genes involved in sex differentiation and gonadal development. Our findings reveal sex‐specific patterns in growth and development, distinct gene expression trajectories and dynamics, and correlations between gene expression, gonadal maturation, and histological features.

Both male and female frogs exhibited increases in body weight and length with age and showed significance for female body weight and body length from Met 1‐2 to Met 5‐6 (Figure 1E,F). The liver and fat body somatic indices in males increased from Met 1‐2 to Met 3‐4 but decreased significantly by Met 7‐8 (Figure 1C,D). In contrast, the fat body somatic index of females increased over time and significantly peaked at Met 5‐6 (Figure 1H). Females did not exhibit significant changes in the liver somatic index across age groups, but it followed a trend similar to the fat body somatic index, reaching a peak at Met 5‐6 (Figure 1G). This pattern may reflect shifts in energy storage and utilization during development. Warne and Crespi (2015) showed that PM female wood frogs (*Lithobates sylvaticus*) tended to proportionally allocate more energy to fat storage faster than males, whereas Morey and Reznick (2001) found that males (*Spea hammondii*) tended to grow faster than females. These differences likely reflect divergent metabolic strategies, with males exhibiting earlier energy utilization potentially linked to earlier sex maturation, while females continue to accumulate reserves to support prolonged somatic growth and future reproductive demands, as seen in other animals (De Block and Stoks 2005).

Gonadal maturation correlated with age in both sexes but manifested differently. Males showed significant increases in testis area and maturity score by Met 7‐8, which were positively correlated with body measurements (body weight, body length, and limb lengths). This indicates that somatic growth and testicular development are closely linked in males. Female gonadal development did not correlate with body measurements, implying that ovarian maturation may be regulated independently of overall body growth after metamorphosis, mostly due to the delayed gonadal maturation compared to males. This is in line with what is observed in females of different anuran tadpoles, which show heterochrony of ovarian differentiation, independent of somatic development (Ogielska and Kotusz 2004).

Our gene expression analysis revealed some clear sex‐specific patterns across *X. tropicalis* juvenile development. In males, genes associated with steroidogenesis and male differentiation (*cyp17* and *amh*) tended to increase in expression with age, with an overall significant effect of sex and stage detected by ANODEV. The progressive upregulation of *cyp17* in males is consistent with its established role in androgen synthesis, which is essential for testicular growth and spermatogenesis (Payne and Hales 2004). In *X. tropicalis*, *cyp17* expression has been reported to be 8‐ to 11‐fold higher in a testis cluster compared to ovarian ones during the early stages of sexual differentiation (NF58, NF66, 1 week PM, 2 weeks PM—Haselman et al. 2015), in agreement with our results and reflecting the early requirement for androgen production in the male gonad. This functional role is further supported by the significant positive correlations of *cyp17* with testis maturity and area (Table 2), as well as with male‐associated genes including *amh*, *cyp26b1*, *dmrt1*, and *sox9* (Figure 7). Moreover, its preferential clustering with males in the PCA (Figure 5A) reinforces the view that *cyp17* serves as a central marker of male differentiation during the pre‐pubertal period in *X. tropicalis*.

Similarly, *amh* and its type II receptor (*amhr2*) play a critical role in male sex differentiation by inhibiting the development of female reproductive structures (Josso and Clemente 2003). Our findings on *amh* are consistent with those of Jansson et al. (2016), who reported sex‐dependent expression of *amh* during gonadal differentiation in *X. tropicalis* from NF53 to 6 days PM. For *amhr2*, a significant increase was observed in females from NF65 to 4 weeks PM compared to males (Jansson et al. 2016). Our results show an upregulation in females at Met 3‐4 followed by a significant downregulation at Met 7‐8. In males, *amhr2* expression showed a gradual increase, reaching significance compared to females at Met 7‐8. This dynamic was confirmed by ANODEV, which detected a significant sex × stage interaction. Correlation analyses further supported these patterns: *amhr2* expression was negatively correlated with ovary area and positively correlated with testis area and testis maturity (Table 2). Both *amh* and *amhr2* also displayed significant positive correlations with each other and several male‐associated transcripts (Figure 7) and emerged as key nodes in the Bayesian network stratified by age (Figure 6A,B). Specifically, *amh* maintained a robust positive association with *esr1* across developmental stages, while *amhr2* was strongly and positively linked to *esr1* and *dmrt1* toward the last stages (i.e., Met 5‐8). Taken together, these results align with earlier observations during the first 4 weeks PM (Jansson et al. 2016) but also reveal a later divergence: A downregulation of *amhr2* in females and a late upregulation in males, indicating a distinct sex‐specific timing and role for this receptor during gonadal maturation.

Genes involved in the retinoic acid pathway, a well‐established trigger for germ cell meiosis initiation in vertebrates (Teletin et al. 2017), were investigated. In tetrapods, while *aldh1a2* encodes the enzyme that synthesizes retinoic acid, *cyp26b1* encodes the cytochrome P450 enzyme that degrades it, and, in developing gonads, a balance between retinoic acid synthesis and degradation determines when germ cells enter meiosis (Feng et al. 2015; Piprek et al. 2013; Wallacides et al. 2009). In the developing gonads of anuran larvae, including *X. laevis*, high levels of ALDH1A2 in ovaries likely promote retinoic acid synthesis and trigger meiosis, whereas in testes, reduced *aldh1a2* and upregulated *cyp26b1* suppress retinoic acid and prevent meiosis (Piprek et al. 2013). In our study, a similar pattern was observed in pre‐pubertal *X. tropicalis*. In females, there was a significant upregulation of *aldh1a2* from Met 5‐6 onward, whereas in males, an increase in *cyp26b1* expression was observed. In both transcripts, ANODEV detected a significant effect of sex, while for *aldh1a2* expression, it also revealed significant effects of stage and the sex × stage interaction. In addition, *aldh1a2* expression was positively correlated with ovarian area and the number of follicular oocytes, whereas *cyp26b1* expression was associated with testicular area and maturation status (Table 2). This is corroborated by the opposite direction of the transcripts in most of PCAs (Figure 5) and by their strong negative correlation (*ρ* = −0.658, *p* < 0.001; Figure 7). The positive correlation between *aldh1a2* and ovarian maturity likely indicates that developing ovaries sustain or enhance retinoic acid synthesis to support oocyte development. Moreover, Piprek et al. (2013) showed that inhibiting retinoic acid in amphibian tadpoles blocks meiotic entry, while exogenous retinoic acid increases oocyte numbers. This suggests that higher *aldh1a2* expression would promote the progression of meiosis, and thus result in more developed gonads and larger gonadal size. In males, *aldh1a2* might remain comparatively low until the initiation of spermatogenesis. Interestingly, our results showed *cyp26b1* levels to be relatively stable in pre‐pubertal *X. tropicalis* males, with a slight increase during the latest developmental stages analyzed. A similar pattern was observed in the Chinese tongue sole (*Cynoglossus semilaevis*), where *cyp26b1* is initially expressed at comparable levels in male and female gonads, with a male‐biased increase at later stages of juvenile development (Cui et al. 2022). Early juvenile parity in *cyp26b1* expression, followed by a potential male upregulation, suggests that the critical suppression of meiosis happens during early differentiation and, by the time juveniles grow, both sexes might retain a similar capacity for retinoic acid catabolism. This aligns with the likelihood that a major wave of oocyte meiosis is occurring in females around 7‐8 weeks PM, while active spermatogenesis is just initiating in males, as indicated by the high maturity scores in the testes (Figure 2D).

The gene *ddx4* is expressed by the germ cell lineage, conserved both in invertebrates and vertebrates (Castrillon et al. 2000; Lasko 2013). It may be considered a germ cell marker present from early developmental stages in PGCs—as in zebrafish (Olsen et al. 1997; Yoon et al. 1997). In humans, the protein is expressed in germ cells from fetal development in both sexes, with the highest levels observed in spermatocytes and mature oocytes (Castrillon et al. 2000). In *X. laevis*, DDX4 expression in adult gonads is more pronounced during the early stages of spermatogenesis and oogenesis (Komiya et al. 1994). Specifically, strong immunohistochemical staining is observed in spermatogonia and spermatocytes, weaker staining in spermatids, and no signal in spermatozoa, whereas during oogenesis, intense staining is detected in early stages and remains detectable up to stage VI oocytes (Komiya et al. 1994). In larval stages, DDX4 was immunolocalised in the cytoplasm of PGCs at NF stage 46 (Komiya et al. 1994), and its functions appear to be essential for germ cell formation, particularly for PGC migration and survival (Shimaoka et al. 2017). In our experiment, *ddx4* mRNA levels increased with age and were consistently higher in females than in males. This observation aligns with findings in early stages of gonad development in *X. laevis*, where the protein expression increases as more mature germ cells are present in the gonad (Komiya et al. 1994). Our data show a sex‐bias that was not observed before in anuran species but has been described in the human fetal gonad, where both protein and transcript levels are higher in the fetal ovary compared to the fetal testis (Castrillon et al. 2000). In *X. tropicalis*, *ddx4* transcripts increased significantly in correlation with gonadal size and maturity, in both ovaries and testes (Table 2). This is corroborated by the PCA analysis, where *ddx4* and gonadal size/maturity take the same direction (Figure 5). This trend may reflect the growing number of developing germ cells in the gonads as they approach puberty.

The helix‐loop‐helix factor *id4* prevents activation of pro‐differentiation genes and thereby maintains cells in an undifferentiated and proliferative state (Oatley et al. 2011; Sun et al. 2015). In the mouse testis, *id4* expression is largely confined to the SSCs and is critical for sustaining the stem cell pool (Sun et al. 2015). Males lacking *id4* gradually lose their germline due to depletion of undifferentiated spermatogonia, confirming the role of ID4 in SSCs maintenance (Oatley et al. 2011). Accordingly, as SSCs initiate differentiation, *id4* is downregulated (Helsel et al. 2017). A similar role is described in females, where *id4* transcript was observed in premeiotic germ cells in the zebrafish ovary (Y. Liu et al. 2022) and in the mouse ovary, where it might contribute to follicular maturation (Best et al. 2014). This role appears conserved in *X. tropicalis*, where *id4* is more expressed in males than in females throughout juvenile development, as also confirmed by the significant effect of sex in ANODEV. Its expression remains relatively stable in males from 1 to 8 weeks PM, consistent with a maintained SSC population during early testicular development. In contrast, *id4* levels tend to drop over time in females, likely reflecting the transition of oogonia into differentiated oocytes at this stage.

In male peripubertal *X. tropicalis*, a ratio between *id4* (marker of SSCs) and *ddx4* (marker of more mature sperm cells) has been proposed by Svanholm et al. 2024 as a molecular endpoint to assess testicular germ cell composition and detect normal versus disrupted spermatogenesis. In our dataset, these two transcripts were negatively correlated (*ρ* = −0.495, *p* < 0.01; Figure 7), consistent with their complementary biological roles. This antagonistic relationship was further captured in the Bayesian networks, where a strong negative conditional dependence was recovered at early juvenile stages (Met 1‐4; Figure 6A) and within the female‐specific network (Figure 6D). Together, these findings reinforce the view that *id4* and *ddx4* represent different germ cell states, and their balance provides a sensitive molecular readout of germline dynamics in tropical clawed frogs in both sexes.

The gene *dmrt1* has emerged as a key regulator of male gonadal development across most vertebrates, showing sexual dimorphic expression in differentiating gonads and being functionally required for testis formation (Matson and Zarkower 2012; Augstenová and Ma 2025). The role of *dmrt1* in the sex determination cascade is particularly well characterized in *X. laevis*, where it interacts with its duplicated variant (*dm‐w*) to either inhibit or promote masculinization (Yoshimoto et al. 2008, 2010). In developing *X. laevis*, DMRT1 appears in germ stem cells of both sexes, but after metamorphosis, it persists exclusively in spermatogonial cells, apparently disappearing from female oogonia (Fujitani et al. 2016). In other anuran species (i.e., *Engystomops pustulosus*, *Glandirana rugosa*), *dmrt1* does not exhibit sexual dimorphism in the gonad‐mesonephros complex (GMC) during early development (Duarte‐Guterman et al. 2012; Matsushita et al. 2007). In *X. tropicalis*, *dmrt1* gradually increases between NF52 and metamorphosis in the GMC of males and females, whereas in adults expression levels in the testes are significantly elevated (up to 2000‐fold) compared to ovaries (Duarte‐Guterman and Trudeau 2011). Our study fills the gap between tadpoles and adults: we observed no sex or stage‐specific differences in juveniles until Met 5‐6 stages; however, at Met 7‐8 *dmrt1* expression tended to—not significantly—increase in males, as previously reported in adults by Duarte‐Guterman and Trudeau (2011). Moreover, *dmrt1* transcript levels positively and significantly correlated with testis area, testicular maturity (Table 2), and male‐associated transcripts (i.e., *amh*, *amhr2* – Figure 7), and clearly associated with male individuals in PCA analyses (Figure 5A).

The transcript *sox9* is a well‐known testis‐determining factor across many vertebrate species (Vining et al. 2021). However, in *X. tropicalis*, *sox9* does not appear to act as a primary sex‐determining gene but rather plays roles in the differentiation of both testes and ovaries. Specifically, the protein and transcript are detected in both sexes from NF66 (El Jamil et al. 2008a, 2008b), corresponding to the onset of metamorphosis. In the adult testis, SOX9 protein and mRNA are confined to Sertoli‐like cells (El Jamil et al. 2008a), consistent with its established role in testis differentiation across vertebrates. In the ovary, SOX9 shows subcellular expression restricted to the cytoplasm of previtellogenic oocytes, and later in the oocyte nucleus in more developed ones, indicating a possible function in oocyte maturation (El Jamil et al. 2008a). This dual expression profile is consistent with our findings, where *sox9* levels did not differ significantly between male and female gonads at any of the post‐metamorphic time points examined. This lack of sexual dimorphism of *sox9* in *X. tropicalis* may reveal a more important role in the structural and functional differentiation of the gonads in both sexes during juvenile development. This interpretation is further reinforced by PCA, where *sox9* does not align with either sex‐specific cluster but remains centrally positioned (Figure 5A). Nonetheless, network‐level analyses highlight potential sex‐dependent interactions, where *sox9* formed a robust positive association within both sexes (*amhr2* in males (Figure 6C) and with *id4* in females (Figure 6D). Moreover, Spearman correlations revealed diverse associations with both male‐ and female‐linked transcripts (Figure 7).

3βHSD is a key enzyme in the steroidogenic pathway that converts pregnenolone into progesterone, and it is crucial for synthesizing all classes of steroids (glucocorticoids, mineralocorticoids, androgens, and estrogens—Simard et al. 2005). In anuran amphibians, 3βHSD mRNA and protein are found in the interrenal glands (homologs of adrenal glands) and gonads from early developmental stages (Hsu et al. 1980; Hsu et al. 1984; Sakurai et al. 2008). During sex differentiation, *3βhsd* expression remains at comparable levels in male and female undifferentiated gonads of *Glandirana rugosa* (Iwade et al. 2008; Maruo et al. 2008; Sakurai et al. 2008). Our findings are consistent with this pattern, showing no sex‐ or age‐related differences in *3βhsd* expression. Although ANODEV detected a relatively weak sex effect (*p* = 0.02), the lack of significance in pairwise comparisons likely reflects a constitutive role of *3βhsd* in steroidogenesis across both male and female post‐metamorphic *X. tropicalis*. As a key enzyme in the biosynthesis of all steroid hormones, 3βHSD is required in both testes and ovaries to support basal hormone production, regardless of sex. During juvenile stages, when sex steroid output is still low, consistent expression levels may suffice for essential functions in gonadal and interrenal tissues. It is important to note that our measurements derive from the GKC, which includes both gonadal and interrenal tissues, and this composite sampling may mask localized and tissue‐specific variation in expression. Nonetheless, *3βhsd* shows significant negative correlations with ovary area (Table 2), with *aldh1a2* and *ddx4*, while being positively correlated with the retinoic acid‐inhibiting gene *cyp26b1* (Figure 7). Moreover, in the Bayesian network, it exhibited a strong negative association with *cyp19* at early juvenile stages and with *ddx4* at later stages, while showing a positive dependence with *amhr2* in females (Figure 6).

Aromatase, encoded by *cyp19*, catalyses the conversion of androgens to estrogens and is a key regulator of ovarian differentiation and estrogen biosynthesis in vertebrates. In the Japanese wrinkled frog, aromatase expression is upregulated in genetic females during gonadal differentiation, while remaining low in males (Maruo et al. 2008). Similarly, in *X. laevis*, *cyp19* is dimorphically expressed in GMC from NF56 through completion of metamorphosis, being 10‐fold higher in females (Urbatzka et al. 2007). In *X. tropicalis*, *cyp19* expression was analyzed in the GMC of developing tadpoles at five different stages from NF48 to NF60 (Navarro‐Martín et al. 2012). Although no clear sex differences were found between the samples at NF48 (classified as undifferentiated gonads), in adult *X. tropicalis* a significant dimorphic expression of aromatase mRNA was described, with ovaries expressing 10,000‐fold more *cyp19* than testes (Navarro‐Martín et al. 2012). In our dataset, *cyp19* showed a high individual variation in males at Met 1‐2 and higher expression compared to females at Met 7‐8, while ANODEV indicated a significant stage effect, and the PCA did not reveal clear segregation by sex. Nonetheless, *cyp19* showed a single and significant positive correlation with *aldh1a2* (Figure 7), and the same association was recovered as a strong positive dependence in the Bayesian network within the Met 5‐8 and male clusters (Figure 6B,C). Even if sexual dimorphism of aromatase expression is likely present during the larval stage (Navarro‐Martín et al. 2012), the juvenile period analyzed here reflects a phase in which females potentially have not yet reached their peak ovarian estrogen production and *cyp19* upregulation. In light of this, caution should be taken when using aromatase transcripts as an ovarian differentiation marker prior to puberty in *X. tropicalis*. Instead, our results point to *cyp17* as a more reliable differentiation marker in juvenile *X. tropicalis*.

Estrogen receptor alpha (ERα, encoded by *esr1*) mediates estrogen signaling across vertebrates, regulating cell differentiation and morphogenesis during development and reproduction, with established roles in ovarian differentiation in mammals and conserved functions in non‐mammalian species (Heldring et al. 2007; Nagahama et al. 2021). While the allotetraploid *X. laevis* retains two *esr1* paralogs (Wu et al. 2003; Iwabuchi et al. 2008), the diploid *X. tropicalis* carries a single *esr1* locus (Takase and Iguchi 2007). Takase and Iguchi (2007) found that in *X. tropicalis*, GMC/GKC *esr1* expression shows a significant peak at NF60, and that 2 weeks PM females display higher expression levels than males, but in adulthood, this trend reverses. Our results with the same animal model show that *esr1* remains expressed in both sexes after metamorphosis, without major stage‐dependent fluctuations, but with a significant sex effect toward males at later stages. This pattern, also recovered in multivariate, correlation, and network analyses—where *esr1* clustered with male‐associated mRNAs and emerged as a central node—indicates that ERα may contribute to the organization of pre‐pubertal testicular maturation.

While the present study provides a comprehensive transcriptional framework of pre‐pubertal gonadal development in *Xenopus tropicalis*, some limitations should be acknowledged. First, our analyses are based exclusively on mRNA expression levels. It is well established that transcript abundance does not necessarily correlate with protein levels, due to post‐transcriptional regulation, translational control, and protein stability mechanisms (see de Sousa Abreu et al. 2009; J. Liu 2008; Marini et al. 2025; Schwanhäusser et al. 2011). Therefore, although the observed transcriptional patterns strongly associate with histological maturation and gonadal phenotypes, they do not directly demonstrate corresponding changes at the protein level. Future studies investigating the spatial distribution, abundance, and functional activity of the encoded proteins within developing gonads will be important to further validate and refine the potential regulatory networks proposed here. Second, sex determination in this study was based solely on phenotypic and histological criteria. In *X. tropicalis*, sex determination is genetically complex, involving three sex chromosomes (Z, W, and a young Y) and heterogametic conditions in both sexes, as outlined in the Introduction. Under such a system, genetic sex composition (i.e., ZW, WW, YZ, YW, or ZZ) may influence transcriptional dynamics independently of, or in addition to, phenotypic sex. Future transcriptomic studies in *X. tropicalis* would therefore benefit from integrating molecular sex genotyping alongside phenotypic sexing, in order to disentangle the respective contributions of chromosomal sex and gonadal phenotype to gene expression patterns.

## 5 Conclusions

This study provides the first integrated characterization of post‐metamorphic gonadal development and sex‐specific gene expression in juvenile *Xenopus tropicalis* during the pre‐pubertal period. By combining morphological, histological, transcriptional, and network‐level analyses, we identified consistent patterns of sexual dimorphism and ontogenesis associated with gonadal maturation.

Our results demonstrate that gonadal development in both sexes progresses markedly within the first 2 months after metamorphosis, with males showing coordinated increases in testis size and maturity score in parallel with somatic growth, while female ovarian development proceeds more independently of body size. Gene expression analyses revealed significant sexual dimorphism, with *cyp17*, *amh*, and *amhr2* correlating with testis size and maturation and/or being expressed at higher levels in males, whereas *aldh1a2* and *ddx4* were preferentially upregulated in females and strongly associated with ovarian size and follicular oocyte formation. Importantly, the decline of *id4* in females, contrasted with its stable expression in males and concurrent with *ddx4* dynamics, indicates divergent strategies in both sexes: in females, the transition of oogonia into meiosis, and in males, the sustained maintenance of the SSC pool.

Across independent analytical approaches, a set of core transcripts emerged as potential regulatory hubs: *dmrt1*, *amhr2*, *cyp17*, *esr1*, and *ddx4*. These genes, consistently associated with histological gonadal size and maturity, may form the backbone of sex‐specific transcriptional networks in the tropical clawed frog. Particularly notable was the recurrent involvement of retinoic acid pathway genes (*aldh1a2* and *cyp26b1*), which defined opposite trajectories in ovaries and testes and likely mediate the transient meiotic wave in females versus a sustained baseline state in males.

Collectively, our findings highlight that sexually dimorphic transcriptional programs are present immediately after metamorphosis and reflect divergent strategies of germ cell proliferation, meiosis initiation, and steroidogenic differentiation. The identification of sex‐specific transcript interactions provides novel insight into the molecular architecture of amphibian puberty onset. This work establishes a framework for future studies on the evolution of vertebrate sex differentiation, and it offers molecular and histological endpoints in a sensitive amphibian model.

## Author Contributions

**Marini Daniele:** methodology, validation, formal analysis, investigation, data curation, writing – original draft, writing – review and editing, visualization. **Roza Mauricio:** formal analysis, data curation, software, writing – review and editing, visualization. **Berg Cecilia:** conceptualization, resources, supervision, project administration, funding acquisition. **Brouard Vanessa:** conceptualization, methodology, validation, formal analysis, investigation, data curation, writing – original draft, writing – review and editing, visualization.

## Ethics Statement

This study was approved by the Uppsala Ethics Committee for Animal Care and Use (5.8.18–09239/2018) and Uppsala University and carried out in accordance with the ARRIVE guidelines and the EU Directive (2010/63/EU) for animal experiments.

## Conflicts of Interest

The authors declare no conflicts of interest.

## Supporting information

Supplementary_Files.

## Data Availability

The data included in this study are available from the corresponding author upon reasonable request.
