# Supplementary material for: Sex‐Specific Gene Expression Ontogeny During Gonadal Development in Post‐Metamorphic Xenopus tropicalis
Source: Mol Reprod Dev. 2026 Apr 6;93(4):e70098. doi: 10.1002/mrd.70098 (PMC13051529; doi:10.1002/mrd.70098)

Supplementary files

Sex-specific gene expression ontogeny during gonadal development in post-metamorphic *Xenopus tropicalis*

Daniele Marini[1,2,*], Mauricio Roza[3], Cecilia Berg[1] and Vanessa Brouard[1]

[1]Department of Organismal Biology, Uppsala University, 754 36 Uppsala, Sweden;

[2] Department of Veterinary Medicine, University of Perugia, 06126 Perugia, Italy;

[3] Science for Life Laboratory, Department of Environmental Science, Stockholm University, 114 18 Stockholm, Sweden;

*Corresponding author: daniele.marini@collaboratori.unipg.it

Supplementary Table 1

MALES	BW	BL	HLL	FLL	LW
BL	0.97****				
HLL	0.92****	0.93****			
FLL	0.86****	0.81****	0.83****		
LW	0.45	0.36	0.26	0.13	
FBW	0.37	0.28	0.23	0.08	0.75***

Supplementary Table 1A : Correlation between body measurements in male post metamorphic frogs.

Morphological data evaluated at dissection : the body weight (BW), body length (BL), fore limb length (FLL), hind limb length (HLL), liver weight (LW), fat body weight (FBW). Numbers represent the r coefficient of Pearson correlation and statistical significance *** ($p < 0.001$) and **** ($p < 0.0001$).

FEMALES	BW	BL	HLL	FLL	LW
BL	0.96****				
HLL	0.89****	0.86****			
FLL	0.68**	0.67**	0.72**		
LW	0.81***	0.75***	0.64**	0.42	
FBW	0.89****	0.83***	0.72**	0.42	0.94****

Supplementary Table 1B : Correlation between body measurements in female post metamorphic frogs.

Morphological data evaluated at dissection : the body weight (BW), body length (BL), fore limb length (FLL), hind limb length (HLL), liver weight (LW), fat body weight (FBW). Numbers represent the r coefficient of Pearson correlation and statistical significance : ** ($p < 0.01$); *** ($p < 0.001$) and **** ($p < 0.0001$).

Supplementary Table 2

<u>Correlation between body measurement data and gonad maturity in male and female post metamorphic frogs.</u>
Gonad maturity evaluated has testis area and testis maturity score in males and ovary area and follicular oocytes number in females. Morphological data evaluated at dissection : the body weight (BW), body length (BL), fore limb length (FLL), hind limb length (HLL), liver weight (LW), fat body weight (FBW). Numbers represent the r coefficient of Pearson correlation and statistical significance : $p < 0.05$ (*), $p < 0.01$ (**), $p < 0.001$ (***) and $p < 0.0001$ (****).

MALES	Testis area	Testis maturity
**Testis maturity**	0.84****	
**BW**	0.78****	0.67**
**BL**	0.80****	0.71***
**HLL**	0.72***	0.70***
**FLL**	0.60**	0.54*
**LW**	0.15	0.02
**FBW**	0.04	0.09

FEMALES	Ovary area	Follicular oocytes
**Follicular oocytes**	0.95****	
**BW**	0.42	0.40
**BL**	0.47	0.44
**HLL**	0.31	0.30
**FLL**	0.05	0.04
**LW**	0.11	0.19
**FBW**	0.23	0.20

Supplementary Table 3

<u>Analysis of deviance (ANODEV) results for candidate genes.</u>
Tables report the output of generalized linear models (gamma distribution with log link) testing for the main effects of sex, developmental stage, and their interaction (sex×stage). Significant effects ($p < 0.05$) are highlighted, indicating transcripts with consistent overall differences across stages, sex and their interaction.

id4

term	df	deviance	df.residual	residual.deviance	statistic	pvalue
NULL	NA	NA	36	18.18375	NA	NA
Stage	3	1.234606	33	16.94914	1.580125	0.216
sex	1	5.805221	32	11.14392	22.289631	5.50e−05 ***
Stage:sex	3	1.056678	29	10.08725	1.352401	0.277

ddx4

term	df	deviance	df.residual	residual.deviance	statistic	pvalue
NULL	NA	NA	36	94.85434	NA	NA
Stage	3	14.568175	33	80.28617	7.6902104	6.28e−04 ***
sex	1	48.236712	32	32.04946	76.3892130	1.28e−09 ***
Stage:sex	3	1.446318	29	30.60314	0.7634789	0.524

dmrt1

term	df	deviance	df.residual	residual.deviance	statistic	pvalue
NULL	NA	NA	35	23.76867	NA	NA
Stage	3	3.6807529	32	20.08791	2.1747559	0.113
sex	1	0.1232411	31	19.96467	0.2184493	0.644
Stage:sex	3	3.4074969	28	16.55718	2.0133038	0.135

sox9

term	df	deviance	df.residual	residual.deviance	statistic	pvalue
NULL	NA	NA	36	22.19293	NA	NA
Stage	3	3.081494	33	19.11144	1.9354288	0.146
sex	1	1.139332	32	17.97211	2.1467798	0.154
Stage:sex	3	1.090806	29	16.88130	0.6851151	0.568

cyp17

term	df	deviance	df.residual	residual.deviance	statistic	pvalue
NULL	NA	NA	36	44.965807	NA	NA
Stage	3	7.639661	33	37.326146	7.633656	6.58e−04 ***
sex	1	26.157833	32	11.168312	78.411821	9.66e−10 ***
Stage:sex	3	1.562544	29	9.605769	1.561316	0.22

amh

term	df	deviance	df.residual	residual.deviance	statistic	pvalue
NULL	NA	NA	33	30.21609	NA	NA
Stage	3	5.359282	30	24.85681	4.6582014	0.0098 **
sex	1	12.093978	29	12.76283	31.5356724	6.67e−06 ***
Stage:sex	3	1.030377	26	11.73245	0.8955867	0.457

amhr2

term	df	deviance	df.residual	residual.deviance	statistic	pvalue
NULL	NA	NA	36	12.259997	NA	NA
Stage	3	0.3187023	33	11.941294	0.4720134	0.704
sex	1	0.2726147	32	11.668680	1.2112660	0.28
Stage:sex	3	4.0202653	29	7.648415	5.9542051	0.00271 **

3ßhsd

term	df	deviance	df.residual	residual.deviance	statistic	pvalue
NULL	NA	NA	36	33.01271	NA	NA
Stage	3	5.5905153	33	27.42219	2.2305081	0.106
sex	1	4.9977447	32	22.42445	5.9820120	0.0208 *
Stage:sex	3	0.6693573	29	21.75509	0.2670607	0.849

esr1

term	df	deviance	df.residual	residual.deviance	statistic	pvalue
NULL	NA	NA	36	17.579171	NA	NA
Stage	3	0.778059	33	16.801112	1.011538	0.402
sex	1	7.292269	32	9.508843	28.441564	1.01e−05 ***
Stage:sex	3	1.162764	29	8.346079	1.511685	0.232

cyp19

term	df	deviance	df.residual	residual.deviance	statistic	pvalue
NULL	NA	NA	24	26.00851	NA	NA
Stage	3	8.9727343	21	17.03578	5.264123	0.00877 **
sex	1	0.6708689	20	16.36491	1.180756	0.292
Stage:sex	2	3.2281313	18	13.13678	2.840820	0.0847

cyp26b1

term	df	deviance	df.residual	residual.deviance	statistic	pvalue
NULL	NA	NA	36	28.23043	NA	NA
Stage	3	1.365498	33	26.86494	0.6494146	0.59
sex	1	6.394027	32	20.47091	9.1227676	0.00523 **
Stage:sex	3	1.308027	29	19.16288	0.6220818	0.606

aldh1a2

term	df	deviance	df.residual	residual.deviance	statistic	pvalue
NULL	NA	NA	36	58.85273	NA	NA
Stage	3	7.153506	33	51.69922	4.877187	0.00725 **
sex	1	24.986797	32	26.71242	51.107231	7.22e−08 ***
Stage:sex	3	6.725980	29	19.98644	4.585705	0.00955 **

Supplementary Table 4

<u>Summaries of Network, weight matrices and edge evidence probability tables for both stratifications (week of metamorphosis; sex) of the Bayesian Network Analysis.</u>

Stratification by week of metamorphosis (Met 1-4; Met 5-8)

Summary of Network

Network	Number of nodes	Number of non-zero edges	Sparsity
Met 1-4	12	23 / 66	0.652
Met 5-8	12	23 / 66	0.652

Weights matrix

	Met 1-4											
Variable	ID4	DDX4	DMRT1	Sox9	Cyp17	AMH	AMH-r2	3bHSD	ESR1	Cyp19	Cyp26b1	Aldh1a2
ID4	0.000	-0.479	0.000	0.000	0.000	0.000	0.000	0.000	0.000	-0.097	0.000	-0.094
DDX4	-0.479	0.000	0.437	-0.248	0.000	0.000	0.000	0.000	-0.268	0.000	-0.164	0.137
DMRT1	0.000	0.437	0.000	0.196	0.000	0.000	0.203	0.000	0.382	0.000	0.000	0.000
Sox9	0.000	-0.248	0.196	0.000	-0.322	0.000	0.000	-0.251	0.212	0.218	0.000	0.000
Cyp17	0.000	0.000	0.000	-0.322	0.000	0.000	0.000	0.000	0.000	0.000	0.333	0.000
AMH	0.000	0.000	0.000	0.000	0.000	0.000	0.000	0.000	0.440	0.000	0.000	0.055
AMH-r2	0.000	0.000	0.203	0.000	0.000	0.000	0.000	0.000	0.000	0.000	0.000	0.000
3bHSD	0.000	0.000	0.000	-0.251	0.000	0.000	0.000	0.000	0.252	-0.529	0.000	0.000
ESR1	0.000	-0.268	0.382	0.212	0.000	0.440	0.000	0.252	0.000	0.000	0.000	0.000
Cyp19	-0.097	0.000	0.000	0.218	0.000	0.000	0.000	-0.529	0.000	0.000	0.168	0.132
Cyp26b1	0.000	-0.164	0.000	0.000	0.333	0.000	0.000	0.000	0.000	0.168	0.000	-0.445
Aldh1a2	-0.094	0.137	0.000	0.000	0.000	0.055	0.000	0.000	0.000	0.132	-0.445	0.000

	Met 5-8											
Variable	ID4	DDX4	DMRT1	Sox9	Cyp17	AMH	AMH-r2	3bHSD	ESR1	Cyp19	Cyp26b1	Aldh1a2
ID4	0.000	-0.126	0.000	0.000	0.000	0.123	0.000	0.196	0.339	0.000	0.000	0.000
DDX4	-0.126	0.000	0.091	0.241	0.000	0.000	0.000	-0.653	0.000	0.000	0.000	0.298
DMRT1	0.000	0.091	0.000	0.143	0.210	0.000	0.495	0.000	0.000	0.000	0.000	0.000
Sox9	0.000	0.241	0.143	0.000	0.000	0.000	0.000	0.000	0.415	0.000	-0.316	0.000
Cyp17	0.000	0.000	0.210	0.000	0.000	0.000	0.000	0.000	0.083	0.000	0.256	0.000
AMH	0.123	0.000	0.000	0.000	0.000	0.000	0.000	0.000	0.342	0.192	0.364	0.000
AMH-r2	0.000	0.000	0.495	0.000	0.000	0.000	0.000	0.000	0.286	0.000	0.000	-0.100
3bHSD	0.196	-0.653	0.000	0.000	0.000	0.000	0.000	0.000	0.000	0.000	0.088	0.000
ESR1	0.339	0.000	0.000	0.415	0.083	0.342	0.286	0.000	0.000	0.000	0.176	0.000
Cyp19	0.000	0.000	0.000	0.000	0.000	0.192	0.000	0.000	0.000	0.000	0.000	0.455
Cyp26b1	0.000	0.000	0.000	-0.316	0.256	0.364	0.000	0.088	0.176	0.000	0.000	0.000
Aldh1a2	0.000	0.298	0.000	0.000	0.000	0.000	-0.100	0.000	0.000	0.455	0.000	0.000

Edge evidence probability table

	Met 1-4											
Variable	ID4	DDX4	DMRT1	Sox9	Cyp17	AMH	AMH-r2	3bHSD	ESR1	Cyp19	Cyp26b1	Aldh1a2
ID4	0.000	0.940	0.450	0.370	0.370	0.250	0.440	0.420	0.300	0.580	0.290	0.540
DDX4	0.940	0.000	0.910	0.790	0.450	0.420	0.210	0.300	0.840	0.260	0.650	0.570
DMRT1	0.450	0.910	0.000	0.510	0.330	0.290	0.570	0.310	0.940	0.450	0.450	0.360
Sox9	0.370	0.790	0.510	0.000	0.750	0.210	0.340	0.680	0.660	0.700	0.480	0.310
Cyp17	0.370	0.450	0.330	0.750	0.000	0.430	0.210	0.340	0.350	0.410	0.700	0.310
AMH	0.250	0.420	0.290	0.210	0.430	0.000	0.290	0.300	0.950	0.410	0.370	0.510
AMH-r2	0.440	0.210	0.570	0.340	0.210	0.290	0.000	0.470	0.220	0.300	0.260	0.160
3bHSD	0.420	0.300	0.310	0.680	0.340	0.300	0.470	0.000	0.770	0.990	0.300	0.490
ESR1	0.300	0.840	0.940	0.660	0.350	0.950	0.220	0.770	0.000	0.360	0.450	0.240
Cyp19	0.580	0.260	0.450	0.700	0.410	0.410	0.300	0.990	0.360	0.000	0.660	0.560
Cyp26b1	0.290	0.650	0.450	0.480	0.700	0.370	0.260	0.300	0.450	0.660	0.000	0.820
Aldh1a2	0.540	0.570	0.360	0.310	0.310	0.510	0.160	0.490	0.240	0.560	0.820	0.000

	Met 5-8											
Variable	ID4	DDX4	DMRT1	Sox9	Cyp17	AMH	AMH-r2	3bHSD	ESR1	Cyp19	Cyp26b1	Aldh1a2
ID4	0.000	0.540	0.310	0.260	0.320	0.640	0.400	0.680	0.930	0.290	0.360	0.430
DDX4	0.540	0.000	0.640	0.770	0.270	0.390	0.270	1.000	0.470	0.310	0.390	0.910
DMRT1	0.310	0.640	0.000	0.540	0.680	0.370	0.920	0.490	0.380	0.170	0.350	0.460
Sox9	0.260	0.770	0.540	0.000	0.300	0.460	0.350	0.460	0.990	0.250	0.860	0.320
Cyp17	0.320	0.270	0.680	0.300	0.000	0.330	0.300	0.340	0.580	0.320	0.740	0.430
AMH	0.640	0.390	0.370	0.460	0.330	0.000	0.280	0.410	0.920	0.670	0.840	0.390
AMH-r2	0.400	0.270	0.920	0.350	0.300	0.280	0.000	0.250	0.910	0.410	0.390	0.530
3bHSD	0.680	1.000	0.490	0.460	0.340	0.410	0.250	0.000	0.250	0.360	0.620	0.270
ESR1	0.930	0.470	0.380	0.990	0.580	0.920	0.910	0.250	0.000	0.280	0.710	0.300
Cyp19	0.290	0.310	0.170	0.250	0.320	0.670	0.410	0.360	0.280	0.000	0.370	0.920
Cyp26b1	0.360	0.390	0.350	0.860	0.740	0.840	0.390	0.620	0.710	0.370	0.000	0.440
Aldh1a2	0.430	0.910	0.460	0.320	0.430	0.390	0.530	0.270	0.300	0.920	0.440	0.000

Stratification by sex (Females [F]; Males [M])

Summary of Network

Network	Number of nodes	Number of non-zero edges	Sparsity
F	12	18 / 66	0.727
M	12	29 / 66	0.561

Weights matrix

	F											
Variable	DDX4	DMRT1	Cyp17	AMH	AMH-r2	3bHSD	Aldh1a2	Cyp26b1	ID4	Sox9	ESR1	Cyp19
DDX4	0.000	0.000	0.000	0.000	0.000	0.000	0.580	0.000	-0.379	0.123	0.000	0.000
DMRT1	0.000	0.000	0.000	0.000	0.000	0.000	0.000	0.000	0.000	0.000	0.182	0.000
Cyp17	0.000	0.000	0.000	0.000	0.000	0.000	0.000	0.000	-0.256	0.000	0.000	0.000
AMH	0.000	0.000	0.000	0.000	0.000	0.000	0.116	0.000	0.000	0.000	0.368	-0.104
AMH-r2	0.000	0.000	0.000	0.000	0.000	0.508	0.000	0.000	0.000	0.000	0.203	0.000
3bHSD	0.000	0.000	0.000	0.000	0.508	0.000	0.000	0.000	0.000	0.000	0.000	0.000
Aldh1a2	0.580	0.000	0.000	0.116	0.000	0.000	0.000	0.000	-0.060	0.263	0.000	0.000
Cyp26b1	0.000	0.000	0.000	0.000	0.000	0.000	0.000	0.000	0.378	-0.395	0.000	0.334
ID4	-0.379	0.000	-0.256	0.000	0.000	0.000	-0.060	0.378	0.000	0.526	0.315	0.000
Sox9	0.123	0.000	0.000	0.000	0.000	0.000	0.263	-0.395	0.526	0.000	0.000	0.325
ESR1	0.000	0.182	0.000	0.368	0.203	0.000	0.000	0.000	0.315	0.000	0.000	0.000
Cyp19	0.000	0.000	0.000	-0.104	0.000	0.000	0.000	0.334	0.000	0.325	0.000	0.000

	M											
Variable	DDX4	DMRT1	Cyp17	AMH	AMH-r2	3bHSD	Aldh1a2	Cyp26b1	ID4	Sox9	ESR1	Cyp19
DDX4	0.000	0.096	0.209	0.000	0.000	-0.172	-0.201	0.000	0.000	0.000	0.116	-0.104
DMRT1	0.096	0.000	0.303	0.000	0.228	0.000	0.000	0.339	0.000	0.000	0.139	0.000
Cyp17	0.209	0.303	0.000	0.000	0.123	0.000	0.135	0.000	0.022	0.152	0.000	0.000
AMH	0.000	0.000	0.000	0.000	0.130	0.000	0.000	0.000	-0.022	0.124	0.405	0.106
AMH-r2	0.000	0.228	0.123	0.130	0.000	0.000	0.000	0.000	0.030	0.294	0.262	0.000
3bHSD	-0.172	0.000	0.000	0.000	0.000	0.000	0.000	0.000	0.142	-0.134	0.000	0.000
Aldh1a2	-0.201	0.000	0.135	0.000	0.000	0.000	0.000	0.000	0.006	0.000	0.000	0.576
Cyp26b1	0.000	0.339	0.000	0.000	0.000	0.000	0.000	0.000	0.000	0.000	0.000	0.000
ID4	0.000	0.000	0.022	-0.022	0.030	0.142	0.006	0.000	0.000	0.079	0.103	0.000
Sox9	0.000	0.000	0.152	0.124	0.294	-0.134	0.000	0.000	0.079	0.000	0.151	0.000
ESR1	0.116	0.139	0.000	0.405	0.262	0.000	0.000	0.000	0.103	0.151	0.000	0.000
Cyp19	-0.104	0.000	0.000	0.106	0.000	0.000	0.576	0.000	0.000	0.000	0.000	0.000

Edge evidence probability table

	F											
Variable	DDX4	DMRT1	Cyp17	AMH	AMH-r2	3bHSD	Aldh1a2	Cyp26b1	ID4	Sox9	ESR1	Cyp19
DDX4	0.000	0.360	0.270	0.470	0.410	0.450	1.000	0.110	0.910	0.510	0.240	0.240
DMRT1	0.360	0.000	0.390	0.260	0.480	0.290	0.420	0.290	0.170	0.220	0.560	0.140
Cyp17	0.270	0.390	0.000	0.380	0.260	0.330	0.200	0.340	0.780	0.160	0.210	0.290
AMH	0.470	0.260	0.380	0.000	0.250	0.430	0.580	0.260	0.400	0.330	0.890	0.530
AMH-r2	0.410	0.480	0.260	0.250	0.000	0.930	0.330	0.230	0.230	0.460	0.650	0.130
3bHSD	0.450	0.290	0.330	0.430	0.930	0.000	0.340	0.500	0.450	0.500	0.220	0.240
Aldh1a2	1.000	0.420	0.200	0.580	0.330	0.340	0.000	0.390	0.560	0.790	0.310	0.210
Cyp26b1	0.110	0.290	0.340	0.260	0.230	0.500	0.390	0.000	0.930	0.880	0.430	0.780
ID4	0.910	0.170	0.780	0.400	0.230	0.450	0.560	0.930	0.000	0.990	0.820	0.180
Sox9	0.510	0.220	0.160	0.330	0.460	0.500	0.790	0.880	0.990	0.000	0.440	0.810
ESR1	0.240	0.560	0.210	0.890	0.650	0.220	0.310	0.430	0.820	0.440	0.000	0.280
Cyp19	0.240	0.140	0.290	0.530	0.130	0.240	0.210	0.780	0.180	0.810	0.280	0.000

	M											
Variable	DDX4	DMRT1	Cyp17	AMH	AMH-r2	3bHSD	Aldh1a2	Cyp26b1	ID4	Sox9	ESR1	Cyp19
DDX4	0.000	0.600	0.630	0.360	0.390	0.530	0.670	0.320	0.490	0.350	0.600	0.510
DMRT1	0.600	0.000	0.810	0.470	0.770	0.290	0.350	0.880	0.280	0.450	0.660	0.500
Cyp17	0.630	0.810	0.000	0.470	0.620	0.280	0.510	0.320	0.530	0.570	0.500	0.450
AMH	0.360	0.470	0.470	0.000	0.590	0.370	0.330	0.390	0.540	0.620	0.900	0.620
AMH-r2	0.390	0.770	0.620	0.590	0.000	0.350	0.430	0.430	0.520	0.910	0.840	0.440
3bHSD	0.530	0.290	0.280	0.370	0.350	0.000	0.500	0.370	0.530	0.550	0.400	0.370
Aldh1a2	0.670	0.350	0.510	0.330	0.430	0.500	0.000	0.330	0.520	0.430	0.360	0.980
Cyp26b1	0.320	0.880	0.320	0.390	0.430	0.370	0.330	0.000	0.480	0.480	0.410	0.300
ID4	0.490	0.280	0.530	0.540	0.520	0.530	0.520	0.480	0.000	0.510	0.710	0.370
Sox9	0.350	0.450	0.570	0.620	0.910	0.550	0.430	0.480	0.510	0.000	0.600	0.470
ESR1	0.600	0.660	0.500	0.900	0.840	0.400	0.360	0.410	0.710	0.600	0.000	0.490
Cyp19	0.510	0.500	0.450	0.620	0.440	0.370	0.980	0.300	0.370	0.470	0.490	0.000

Supplementary Fig. 1 : Edge Evidence Plot showing the strength of connections between transcripts from the gonad/kidney complex in post-metamorphosis frogs, stratified by age (A, B) and sex (C, D). The plots indicate included edges ($BF_{10} > 10$; blue lines) and excluded edges ($BF_{10} < 10$; grey lines) among nodes.

(A) Plot based on relative gene expression data from younger froglets, covering the first to fourth weeks post-metamorphosis (Met1-4). (B) Plot from mRNA data for older frogs, covering the fifth to eighth weeks post-metamorphosis (Met5-8). (C) Plot from gene expression data for male frogs. (D) Plot from gene expression data for female frogs. Germ cells genes: *id4*, *ddx4* and *dmrt1*; steroidogenic/signalling genes: *cyp17*, *3βhsd*, *cyp19* and *esr1*; male differentiation genes: *amh*, *amhr2*, *sox9*; retinoic pathway genes: *aldh1a2* and *cyp26b1*.

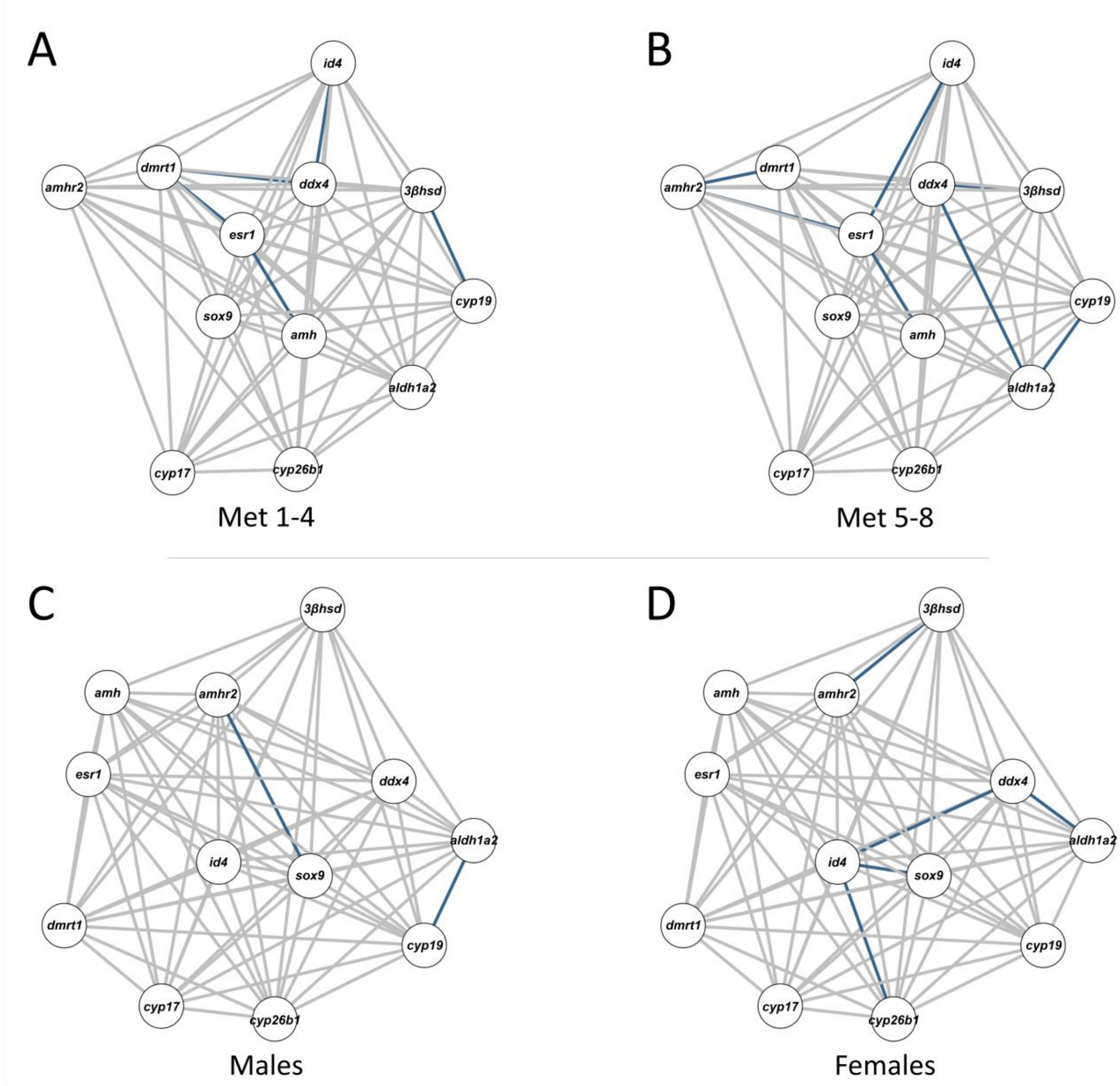

Supplementary Fig. 2 : Centrality plots for transcripts from the gonad/kidney complex in post-metamorphosis frogs stratified by age (A) and sex (B). The plots display four centrality metrics—Closeness, Betweenness, Strength, and Expected Influence—across each transcript. Data are separated by groups within age and sex. Higher centrality values suggest greater importance of nodes within the network.

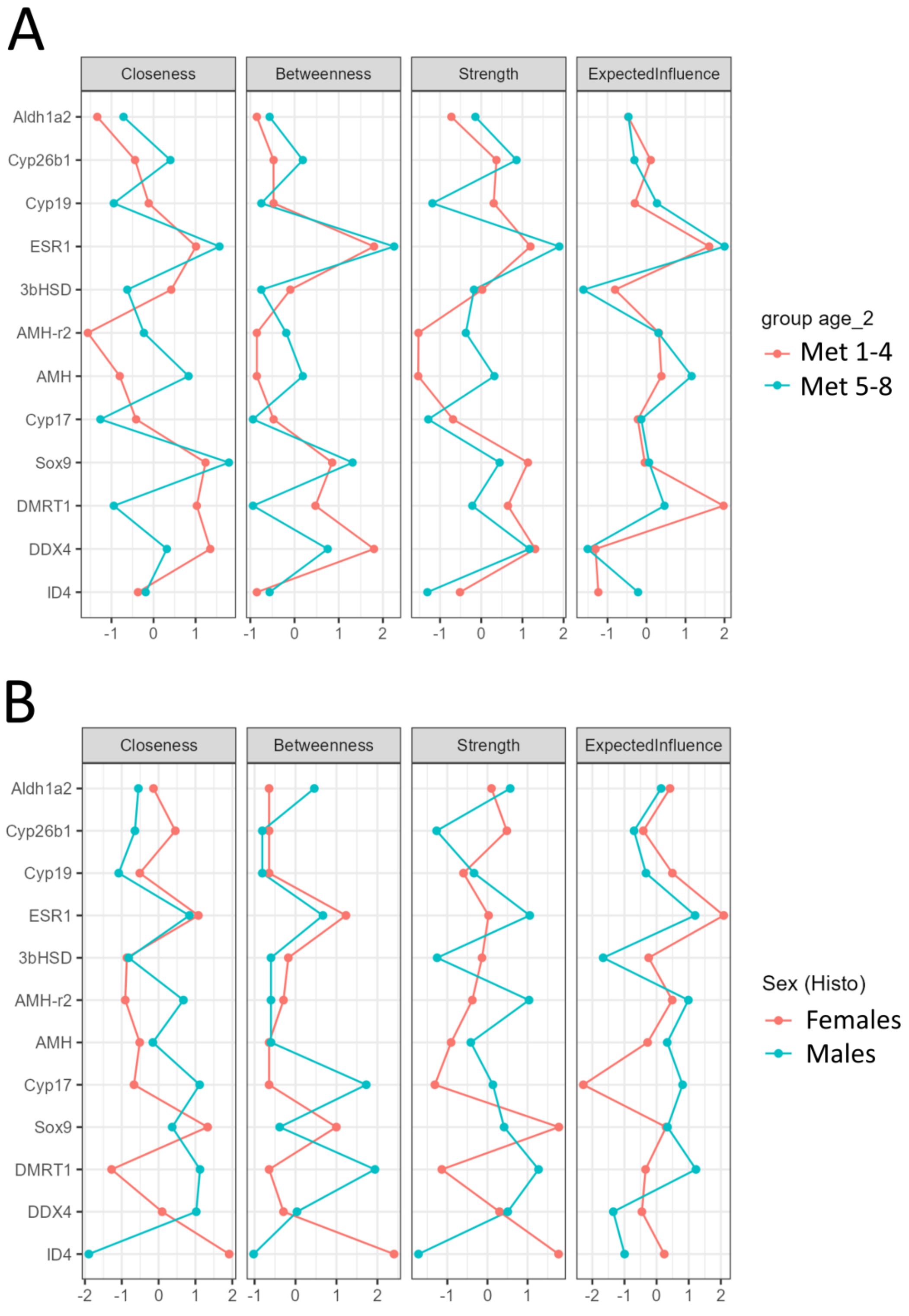

Supplement: Supplementary file 1 — Supplementary_Files. [file MRD-93-e70098-s001.pdf]
